# Autolysosomes and caspase-3 control the biogenesis and release of immunogenic apoptotic exosomes

**DOI:** 10.1038/s41419-022-04591-5

**Published:** 2022-02-11

**Authors:** Déborah Beillevaire, Francis Migneault, Julie Turgeon, Diane Gingras, Annie Karakeussian Rimbaud, Geneviève Marcoux, Crysta Spino, Nicolas Thibodeau, Eric Bonneil, Pierre Thibault, Éric Boilard, Mélanie Dieudé, Marie-Josée Hébert

**Affiliations:** 1 Centre de Recherche, Centre Hospitalier de l’Université de Montréal (CRCHUM), Université de Montréal, Montréal, QC Canada; 2 Canadian Donation and Transplantation Research Program (CDTRP), Edmonton, AL Canada; 3 Département de Médecine, Université de Montréal, Montréal, QC Canada; 4 Département de Pathologie et Biologie Cellulaire, Université de Montréal, Montréal, QC Canada; 5 Département de Microbiologie et Immunologie, Centre de Recherche, Centre Hospitalier Universitaire (CHU) de Québec-Université Laval, Québec, QC Canada; 6 Institute for Research in Immunology and Cancer (IRIC), Montréal, QC Canada; 7 Département de Microbiologie, Infectiologie et Immunologie, Faculté de Médecine, Université de Montréal, Montréal, QC Canada

**Keywords:** Proteomics, Macroautophagy, Apoptosis, Lysosomes

## Abstract

Apoptotic exosome-like vesicles (ApoExos) are a novel type of extracellular vesicle that contribute to the propagation of inflammation at sites of vascular injury when released by dying cells. ApoExos are characterized by the presence of the C-terminal perlecan LG3 fragment and 20S proteasome, and they are produced downstream of caspase-3 activation. In the present study, we assessed the relative roles of autophagy and caspase-3-mediated pathways in controlling the biogenesis and secretion of immunogenic ApoExos. Using electron microscopy and confocal immunofluorescence microscopy in serum-starved endothelial cells, we identified large autolysosomes resulting from the fusion of lysosomes, multivesicular bodies, and autophagosomes as a site of ApoExo biogenesis. Inhibition of autophagy with ATG7 siRNA or biochemical inhibitors (wortmannin and bafilomycin) coupled with proteomics analysis showed that autophagy regulated the processing of perlecan into LG3 and its loading onto ApoExos but was dispensable for ApoExo biogenesis. Caspase-3 activation was identified using caspase-3-deficient endothelial cells or caspase inhibitors as a pivotal regulator of fusion events between autolysosomes and the cell membrane, therefore regulating the release of immunogenic ApoExos. Collectively, these findings identified autolysosomes as a site of ApoExo biogenesis and caspase-3 as a crucial regulator of autolysosome cell membrane interactions involved in the secretion of immunogenic ApoExos.

## Introduction

Autophagy is generally viewed as an autodigestive process aimed at providing energy to cells exposed to stress or facing reduced access to nutrients. Mounting evidence shows that, in addition to its degradative function, autophagy contributes to the secretion of various types of mediators, some of which play important roles in shaping the immune response. One of the first indications that autophagy contributes to the secretion of functionally active mediators comes from the observation that neutrophils and monocytes release IL-1β through an unconventional autophagy-mediated secretory pathway [1, 2]. Our group also showed that the protein constituents of autophagosomes are released by endothelial cells downstream of caspase activation induced by serum starvation [3]. In this system, serum-starved endothelial cells mount an autophagic response leading to the formation of a network of large autophagic vacuoles that fuse with the cell membrane once caspases are activated [3]. Autophagy has also been implicated in the secretion of exosomes and nanosized membrane-bound extracellular vesicles (EVs), containing DNA, RNAs, lipids and proteins, thereby acting as a mediator of intercellular communication [4–6]. The LC3-conjugation machinery at play during autophagy has recently been found to regulate the loading of RNA-binding proteins and small noncoding RNAs into membrane-bound vesicles that, in turn, are released by autophagic cells [7].

Crosstalk between autophagy and regulated cell death programs is complex and occurs at multiple molecular levels [8, 9]. Autophagy can both offset the development of cell death and, in certain cases, behave as a regulated type of cell death. Caspase-3, a key effector of apoptosis, regulates the production of different types of EVs with the best studied being apoptotic bodies. Our group showed that, in addition to classical apoptotic bodies released through budding of the cell membrane, caspase-3 activation triggers the release of smaller EVs (30 to 100 nm) with strong immunogenic activity [10, 11]. EVs, also named apoptotic exosome-like vesicles (ApoExos), lack the usual markers of apoptotic bodies, such as histones and HMGB1, but they carry functionally active 20S proteasomes and perlecan/LG3. ApoExos are produced in association with vascular injury and foster the production of anti-LG3 autoantibodies, which have been shown to aggravate ischemia–reperfusion injury and transplant rejection in animal models and transplant patients [10–14]. Ischemia–reperfusion injury concomitantly induces autophagy and apoptosis of vascular cells, but the relative contributions of both pathways to ApoExo biogenesis remain to be characterized. In the present study, we aimed to delineate the relative contributions of caspase-3-mediated pathways and autophagy in ApoExo biogenesis and release.

## Materials and methods

### Cell culture and conditioned medium preparation

Human umbilical vein endothelial cells (HUVECs) were obtained from Cell Application (200P-05N) and cultured in EGM-2MV complete medium (Lonza, CA94045-820) or M200 complete medium (Thermo Fisher, M200500; S00310) until they reached ~95–100% confluence. To generate conditioned medium, cells were exposed to serum-free medium (apoptotic stress; RPMI 1640, Invitrogen, 11875-093) alone or in the presence of hydrogen peroxide (necrotic stress; 15 mM, Fischer, H325-500), bafilomycin A1 (lysosomal inhibitor; 20 nM, Sigma-Aldrich, B1793, Baf), wortmannin (PI3K3 inhibitor; 100 nM, Sigma-Aldrich, W3144, Wort), ZVAD-fmk (pancaspase inhibitor; 50 μM, R&D Systems), DEVD (caspase-3/-7 inhibitor; 100 μM, R&D Systems), or vehicle (dimethyl sulfoxide, Sigma-Aldrich, D2650) for 1 to 4 h. Preincubation for 1 h in complete medium with wortmannin was applied before serum starvation. Cells were exposed to EGM-2MV vesicle-free complete medium to produce conditioned medium from normal cells.

### siRNA transfection

ATG7 silencing was performed with Magnet Assisted Transfection (IBA GmbH, 7-2021-020) according to the manufacturer’s instructions. Briefly, siCTL (Dharmacon ThermoScientific, D-001810-01-05) or siATG7 (Dharmacon ThermoScientific, L-020112-00-0005) was mixed with MATra-Si reagent. After incubation for 15 min at 37 °C on a magnetic plate, cells were incubated at 37 °C for 30 min. The medium was then replaced with fresh medium containing serum. After 48 h of transfection, HUVECs were exposed to serum starvation.

### Aortic murine cell isolation

Murine ECs (mECs) were isolated from the aortas of C57BL/6 (wild-type; WT) or caspase-3−/− mice and grown in Dulbecco’s modified Eagle’s medium supplemented with endothelial cell growth supplements (ECGS; Alfa Aesar, Haverhill, MA), 10% fetal bovine serum (FBS; Invitrogen, Carlsbad, CA), 10% newborn calf serum (Invitrogen), heparin (12.6 U/mL, Sandoz, Holzkirchen, Germany), 1% penicillin–streptomycin, and 1% amphotericin B. To generate conditioned media, cells were exposed for 9 h to serum‐free media RPMI‐1640 (Invitrogen) alone or in the presence of Baf (20 nM), ZVAD (50 μM), or vehicle (DMSO). In previous work, we demonstrated that this system leads to the release of active mediators by apoptotic ECs downstream of caspase‐3 activation without cell membrane permeabilization [10, 11].

### Screening for apoptosis by fluorescence microscopy

Fluorescence microscopy of unfixed/unpermeabilized adherent cells stained with 1 µg/mL 2′-(4-ethoxyphenyl)-5-(4-methyl-1-piperazinyl)-2.50-bi-1H-benzimidazole (Hoechst 33342, HO; Sigma-Aldrich, B-2261) and 5 µg/mL propidium iodide (PI, Invitrogen, P-3566) was performed as previously described [15].

### Screening for apoptosis by flow cytometry

Briefly, cells were harvested with trypsin/EDTA 0.05% (Lonza, R001-100) after 4 h of treatment followed by centrifugation at 300 × *g* for 5 min at room temperature. Cells were incubated with 5 µg/mL PI and 5 μL of Annexin V (BD Pharmingen, 556419) for 15 min before FACS analysis. Data were processed and analyzed using FACSDiva software (BD Biosciences).

### Analysis of caspase-3 activation

Caspase-3 activity was evaluated using a fluorometric assay kit (BioVision Inc., K105) according to the manufacturer’s instructions. The samples were read on a microplate reader at excitation and emission wavelengths of 400 and 505 nm, respectively.

### Immunoblotting

Proteins were extracted, separated by electrophoresis, and transferred onto nitrocellulose membranes or polyvinylidene difluoride (for caspase-3 detection, Bio–Rad, 162–0175). Membranes were stained with Ponceau S Red (Sigma, P-3504) as a loading control. The antibodies used for blotting were antibodies against PARP (Cell Signaling Technology, 9542), LC3 (Novus, NB600-1384), p62 (Cell Signaling Technology, 8025), beta-actin (Sigma-Aldrich, a5441), GM130 (Abcam, ab52649), CD82 (Abcam, ab66400), TSG101 (Abcam, ab125011), tubulin (Calbiochem, cp06), TCTP (Santa Cruz Biotechnology, SC-30124), syntenin-1 (Santa Cruz Biotechnology, SC-100336 or SC-515538), LAMP2 (Abcam, ab25631), 20S proteasome α3 (Santa Cruz Biotechnology, SC-67340), perlecan/LG3 (Santa Cruz Biotechnology, SC-25848), cleaved caspase-3 (Cell Signaling, 9661L), histone H3 (Cell Signaling, 9715S), ATG7 (R&D Systems, MAB6608), pSer473-AKT (Cell Signaling, 9271S), and total AKT (Cell Signaling, 9272L). Densitometric analysis was conducted with AlphaImager, version 3.2 (Alpha Innotech Corporation, San Leandro, CA, USA). Data are expressed as arbitrary units.

### Apoptotic exosome-like vesicle (ApoExo) isolation

Conditioned serum-free medium from HUVECs was fractionated using sequential centrifugation as previously described [10, 11, 16, 17]. Briefly, the medium was centrifuged at 1200 × *g* for 15 min at 4 °C to pellet cell debris. Protease inhibitor (Sigma-Aldrich, P1860) was added to the supernatant for immunoblotting experiments. The supernatant was then centrifuged at 50,000 × *g* for 15 min at 4 °C to pellet apoptotic bodies and then centrifuged using an ultracentrifuge at 200,000 × g for 18 h at 4 °C to pellet ApoExos.

### Flow cytometric analyses of extracellular vesicles

HUVECs were stained using CellTracker green CMFDA dye (10 mM, Thermo Fischer Scientific, C7025) in SS medium for 15 min at 37 °C before conditioning. After treatment, the supernatant was harvested and centrifuged at 1200 × *g* for 15 min at 4 °C to remove cell debris. Flow cytometric analyses were performed as previously described [18–20]. In brief, fluorescence was used as a trigger signal, and positive fluorescent events were plotted on a SSC/FSC-PMT graph. The ApoExo gate of detection was determined based on the acquisition of 100 nm, 500 nm and 1 µm fluorescent silica particles (Kisker Biotech GmbH & Co. Steinfurt, Germany). Conditioned medium was labeled for 15 min with APC probe-conjugated annexin V (diluted 1:30 in annexin-V binding buffer; BD Biosciences; 550474) at room temperature in the dark. To quantitatively process the data, a known number of polystyrene microspheres (3 μm diameter; Polysciences) were added to each tube as previously described [18], and 10,000 beads were acquired at low speed.

### Electron microscopy

HUVECs or mECs were fixed with 1% glutaraldehyde in phosphate buffer (pH 7.2) (for ultrastructural study) and with 4% paraformaldehyde/0.5% glutaraldehyde (for immunochemistry study). Cells were washed and scraped off the plates in phosphate buffer and pelleted by centrifugation at 10,000 RPM. For the ultrastructural study, the pellets were postfixed with 1% OsO_4_ in phosphate buffer for 1 h at 4 °C, dehydrated in a graded series of ethanol, and embedded in Epon according to routine techniques (Luft 1961). Ultrathin sections were obtained using a Reichert Ultracut ultramicrotome and mounted on naked nickel grids. For immunogold labeling, the pellets were dehydrated in a graded series of methanol and embedded in Lowicryl K4M resin at −20 °C. Ultrathin sections were obtained using a Reichert Ultracut ultramicrotome and mounted on formvar-carbon-coated nickel grids. Immunocytochemistry was performed on grid-mounted tissue sections that were incubated overnight at 4 °C on a drop of anti-perlecan/LG3 antibody (Santa Cruz Biotechnology, SC-25848) diluted 1/10 in PBS. Grids were then incubated on a drop of anti-rabbit 10 nm gold conjugated complex for 30 min at room temperature. Sections were stained with 3% aqueous uranyl acetate and lead citrate, and examination was performed with a Philips CM100 transmission electron microscope. Electron micrographs were captured using an AMT XR80 digital camera. The cytoplasmic area occupied by compartments (autophagosomes, lysosomes, multivesicular bodies, and autolysosomes) was assessed in relation to the cell cytoplasm (nuclei were not included in the evaluation) using ImageJ software.

### Confocal microscopy

HUVECs were cultured on gelatin-coated glass coverslips. After treatment, cells were fixed in 4% paraformaldehyde for 20 min at room temperature and permeabilized for 5 min with Triton X100 (Sigma-Aldrich, T8787). Coverslips were then blocked for 1 h in 5% goat serum/3% BSA in PBS (Sigma-Aldrich, G9023) and incubated overnight in a humidified chamber using 1:200 perlecan/LG3 antibody (Santa Cruz Biotechnology, SC-25848) and 1:100 Lamp2 antibody (Abcam, ab25631) in blocking solution. Fixed cells were subsequently washed with PBS, incubated with 1:800 goat anti-rabbit AlexaFluor-488 (Molecular Probes, A-11008) and 1:800 goat anti-mouse AlexaFluor-594 (Molecular Probes, A-11020) in blocking solution for 1 h at room temperature, and then sealed using Prolong Gold DAPI (Invitrogen, P36935). Confocal images were acquired on a Leica TCS-SP5 inverted confocal microscope using a HCX PL APO 100x/1.4 oil objective. An excitation system was performed using a 405 nm diode laser for DAPI, the 488 nm line of an argon laser for AlexaFluor-488, and a 561 nm diode laser for AlexaFluor-594 using sequential acquisition at a 400 Hz scan speed. Detection bandwidths were 428–492 nm for DAPI, 504–571 nm for AlexaFluor-488, and 595–765 nm for AlexaFluor-594. The final images are 12 bits, 2048 × 2048 for z-stack, and a single plane. Z-stacks were deconvolved using ImagePro Analyzer software. Imaris (Bitplane) software was used for 3D rendering, statistical analysis, and measurement.

### Animal studies

Adult C57BL/6 and BALB/c mice (20–22 g; Charles River; Kingston, NY, USA) were housed in sterilized, ventilated cages in a specific pathogen-free animal facility under a standard 12 h light/12 h dark cycle and fed a normal diet ad libitum. All animal experiments and methods were performed following the relevant guidelines and regulations approved by the CRCHUM’s Comité Institutionnel de Protection des Animaux (CIPA).

### Aortic transplantation procedures

Mice were anesthetized with 2% isoflurane by inhalation. Aortic transplantation was performed as described previously [10, 11]. Briefly, 1 mL of 50 μL/mL heparinized saline was injected into the vena cava to flush the aorta. A 6-mm segment of abdominal aorta measured from below the renal arteries to just above the aortic bifurcation was excised and soaked in ice-cold 0.9% normal saline. When mentioned, warm ischemia was induced by clamping the aorta for 15 min before excision from the donor. The grafts were then excised and sutured orthotopically with end-to-end anastomoses using 11-0 nylon interrupted sutures.

### Injection of murine apoptotic endothelial membrane vesicles

Conditioned serum-free medium from 1 × 10^4^ mECs from C57BL/6 mice was fractionated using sequential centrifugation as follows: a first centrifugation at 1200 × *g* for 15 min at 4 °C to pellet cell debris; a second centrifugation at 50,000 × *g* for 15 min at 4 °C to pellet apoptotic bodies; and a final ultracentrifugation at 200,000 × *g* for 18 h at 4 °C to pellet ApoExos. Pellets containing ApoExos were resuspended in half of the initial volume of conditioned medium. Transplanted mice received tail vein (150 μL) intravenous injections of resuspended ApoExo preparations every other day for 3 weeks for a total of eight doses.

### Assessment of circulating levels of anti-perlecan/LG3

Anti-LG3 titers were measured using a locally developed ELISA. Recombinant perlecan fragment LG3 was produced and purified as previously described [21]. The purity of the recovered LG3 protein was assessed by reducing SDS–PAGE and Coomassie Blue R-250 staining. Recombinant mouse LG3 (5 ng/μL) was first coated onto 96-well Immulon II HB plates (0.5 μg per well; Thermo Electron). Of note, mouse and human LG3 fragments are 3 highly homologous at the amino acid level (87%). After dilution of the sera (1:100), 100 μL of diluted sera was added to each well. The plates were washed, and bound IgG was detected using horseradish peroxidase coupled with anti-mouse IgG (1:5000) (Amersham). Reactions were revealed with 100 μL of tetramethylbenzidine substrate (BD Biosciences) and stopped with 50 μL of 1 M H_2_SO_4_ sulfuric acid. Spectrophotometric analysis was performed at 450 nm, and the results are expressed as optical density × 1000.

### Proteomic analysis of purified ApoExos

ApoExos produced by bafilomycin-treated cells or control cells were isolated as described. Samples were reconstituted in 50 mM ammonium bicarbonate with 10 mM Tris (2-carboxyethyl)phosphine hydrochloride (TCEP; Thermo Fisher Scientific) and vortexed for 1 h at 37 °C. Chloroacetamide (Sigma-Aldrich) was added for alkylation to a final concentration of 55 mM. Samples were vortexed for another hour at 37 °C. One microgram of trypsin was added, and digestion was performed for 8 h at 37 °C. Samples were dried down and solubilized in 5% acetonitrile (ACN) and 0.2% formic acid (FA). The samples were loaded on a 1.5 µL precolumn (Optimize Technologies, Oregon City, OR, USA). Peptides were separated on a homemade reversed-phase column (150-μm i.d. by 200 mm) with a 56-min gradient from 10 to 30%, 0.2% FA, and a 600-nL/min flow rate on an Easy nLC-1000 connected to a Q-Exactive Plus (Thermo Fisher Scientific, San Jose, CA, USA). Each full MS spectrum acquired at a resolution of 70,000 was followed by tandem-MS (MS–MS) spectra acquisition on the 15 most abundant multiply charged precursor ions. Tandem-MS experiments were performed using higher-energy collision dissociation (HCD) at a collision energy of 27%. The data were processed using PEAKS X (Bioinformatics Solutions, Waterloo, ON) and a UniProt/SwissProt mouse database including isoforms (released on 03/10/2015). Mass tolerances of precursor and fragment ions were 10 ppm and 0.01 Da, respectively. The fixed modification was carbamidomethyl (C). The various selected posttranslational modifications included oxidation (M), deamidation (NQ), and phosphorylation (STY).

### Statistical analyses

All data are expressed as the mean ± SEM from at least three independent experiments unless specified otherwise. Data were analyzed using a one-sample *t* test, Student’s *t* test, Mann–Whitney test, or Kruskal–Wallis test accordingly with GraphPad Prism 8 software (GraphPad Software, Inc.). *P* values <0.05 were considered significant. Nonsignificant analyses are represented by ns.

## Results

### Large autolysosomes contribute to serum-starved endothelial cell extracellular membrane vesicle biogenesis

**Fig. 1:**
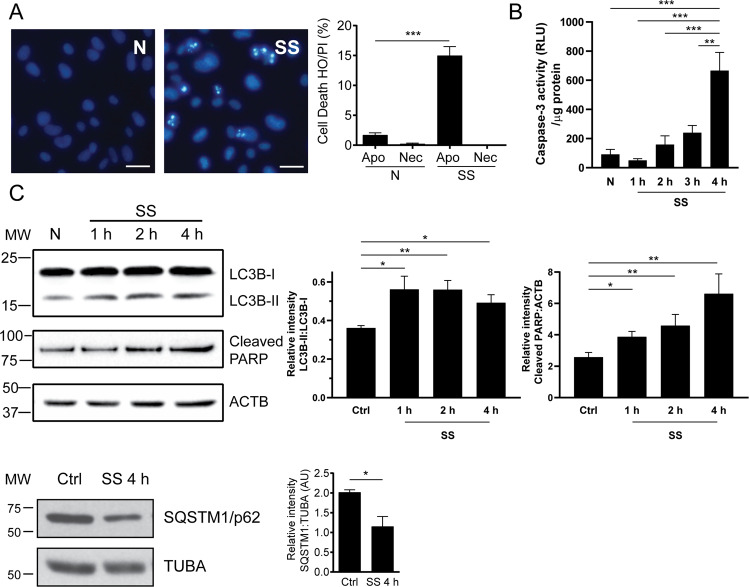
Serum starvation induces autophagy and apoptosis in endothelial cells. **A** Evaluation by Hoechst 33342 and propidium iodide (HO/PI) staining of apoptosis and necrosis in HUVECs exposed to normal medium (N) or serum-starved (SS) for 4 h. *P* values obtained by unpaired *t* test. *n* = 10 for each condition. Scale bar = 25 μm. **B** Quantification of caspase-3 activity in HUVECs exposed to N or SS HUVECs for 1, 2, 3, and 4 h. *P* values obtained by one-way ANOVA. *n* = 4 for each condition. **C** Immunoblot and densitometric analysis of PARP, LC3, and SQSTM1/p62 in HUVECs exposed to N or SS for 1, 2, or 4 h. β-actin (ACTB) was used as a loading control. *P* values obtained by unpaired *t* test (*P < 0.05; **P < 0.01; ***P < 0.001). *n* = 3 for each condition. All values are expressed as the mean ± SEM.

Serum starvation of endothelial cells for up to 4 h promoted an apoptotic response without necrotic features (Fig. 1). Caspase-3 was activated concomitantly with PARP cleavage and chromatin condensation (Fig. 1A–C). Consistent with our previous work [3, 15], activation of autophagy in endothelial cells preceded the development of apoptosis with evidence of LC3 lipidation after 1 h of serum starvation followed by SQSTM1/p62 degradation (Fig. 1B, C) [3]. Upon caspase-3 activation, serum-starved endothelial cells released, in addition to classical apoptotic bodies, smaller EVs reminiscent of exosomes. Using small particle flow cytometry and electron microscopy, we have previously shown that apoptotic exosome-like vesicles (ApoExos) are smaller in size than apoptotic bodies (30 to 100 nm compared to more than 500 nm) [10, 16]. The apoptotic bodies, but not ApoExos, showed increased expression of GM130, histone H3, and tubulin. The ApoExos expressed several exosome markers, such as TSG101, syntenin-1, and TCTP (Fig. S1), but unlike classical exosomes produced by normal endothelial cells, they also expressed a set of specific markers, including LAMP2, LG3, and the active 20S proteasome. The ApoExos also lacked several classical exosome markers, such as CD82. Collectively, these results and our previous work confirmed that ApoExos represent a distinct type of extracellular vesicle released by apoptotic cells.

As caspase-3 is pivotal to the release of ApoExos [10], we investigated whether other death or stress pathways also contribute to ApoExo release. Endothelial cells were induced to undergo necrosis through concomitant exposure to H_2_O_2_ and serum starvation [22]. Consistent with the induction of necrosis, H_2_O_2_ treatment led to cell membrane permeabilization in the absence of chromatin condensation and caspase-3 cleavage (Fig. S2A–C). LG3 was not recovered in medium conditioned by necrotic endothelial cells (Fig. S2D), further supporting a pivotal role for caspase-3 activation in LG3 secretion.

**Fig. 2:**
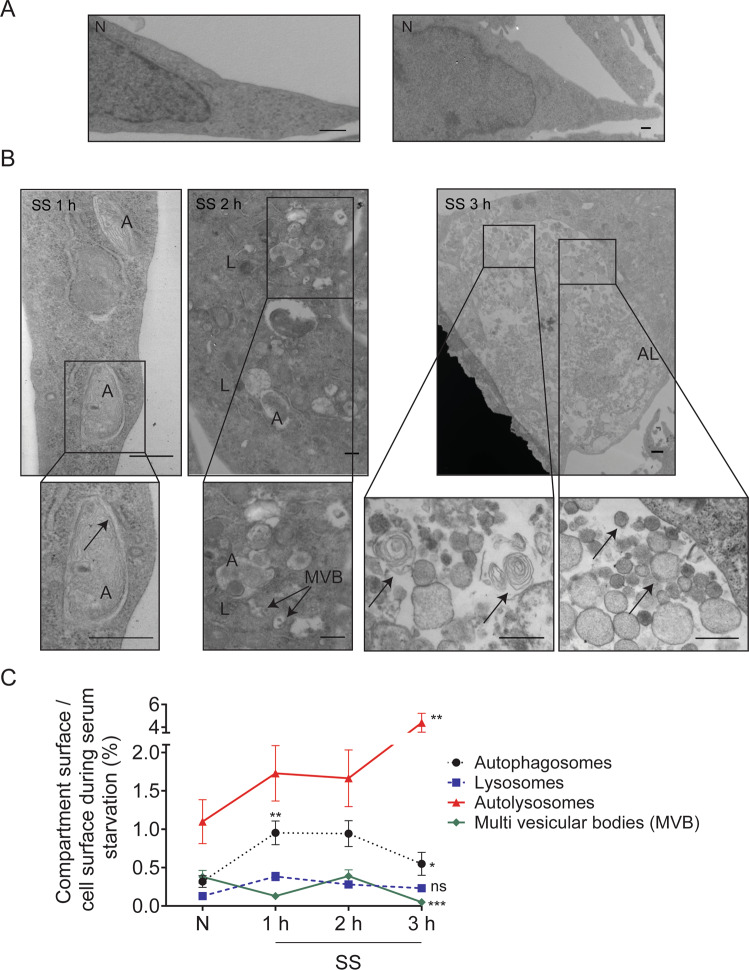
Serum-starved human endothelial cells display large vacuolar networks containing membrane vesicles. **A** Electron micrographs of normal (N) HUVECs representative of two independent experiments. **B** Representative electron micrographs of HUVEC exposed to SS for 1, 2, or 3 h showing autophagosome formation (A; arrow), fusion between lysosomes (L) and autophagosomes (A) with multivesicular bodies (MVB), and large autolysosome (AL) formation containing late multilamellar autophagosomes (left panel, arrows), degraded materials, and ApoExo-size vesicles (right panel arrow) (representative of two independent experiments). **C** Quantification of the cytoplasmic surface occupied by autophagosomes, lysosomes, multivesicular bodies, and autolysosomes on the cell surface in HUVECs exposed to N or SS for 1, 2, or 3 h. Autophagosomes are characterized by a double membrane encapsulating cytoplasmic material. Multivesicular bodies are characterized by the internal budding of the endosomal membrane forming intraluminal vesicles. Lysosomes are characterized by a single membrane containing electron-dense material. Autolysosomes, formed by the fusion between an autophagosome or an MVB with a lysosome, are enclosed by a single membrane and contain cytoplasmic material and organelles in various stages of degradation. Here, autolysosomes were also found to contain an array of small membrane vesicles. Fusion events between autophagosomes or MVBs and lysosomes were observed, resulting in the formation of large autolysosomes. All values are expressed as the mean ± SEM. *P* values were obtained by the Kruskal–Wallis test (*P < 0.05; **P < 0.01; ***P < 0.001). *n* = 50 cell profiles from two independent experiments. Scale bar = 500 nm.

**Fig. 3:**
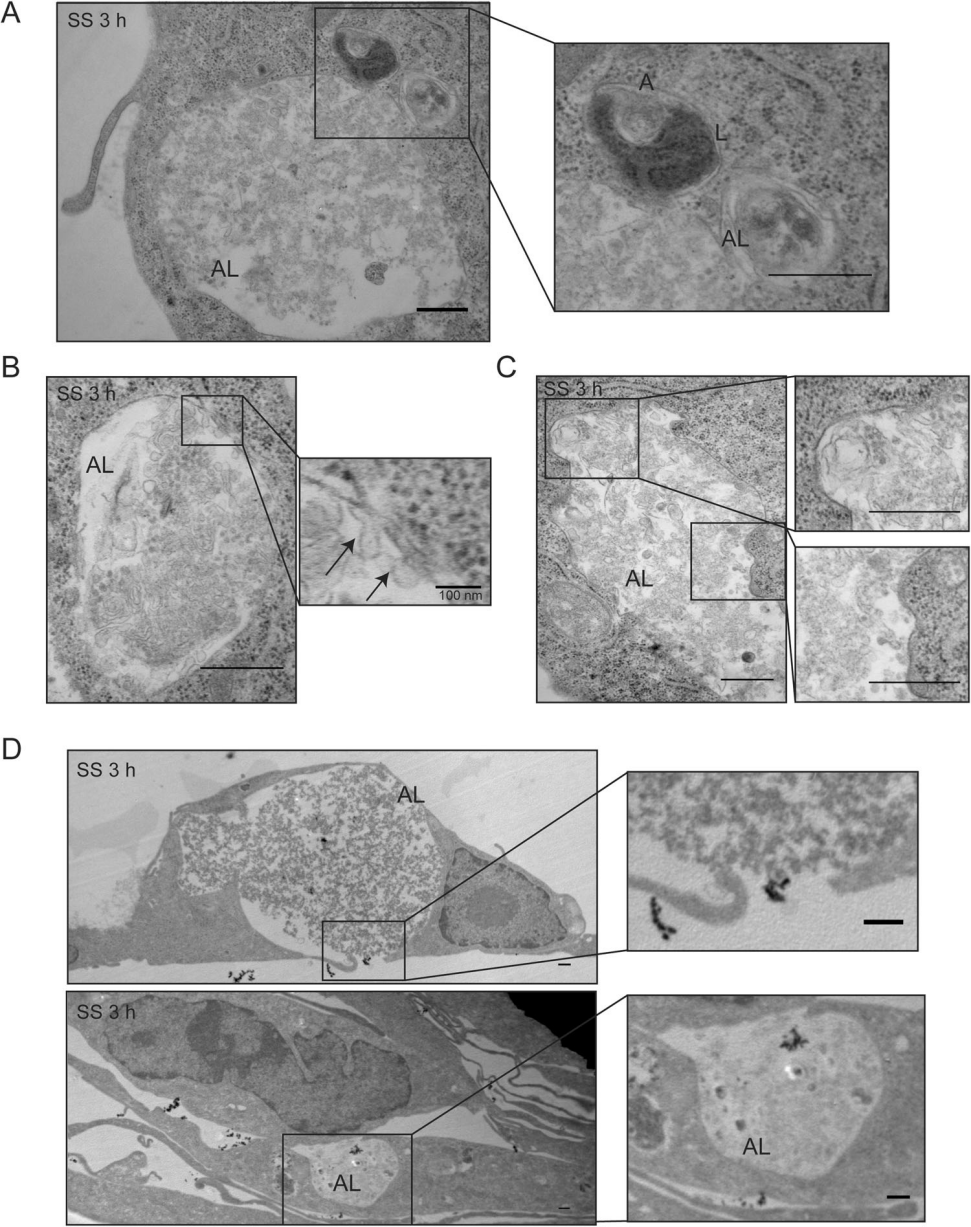
Serum-starved human endothelial cells display large autolysosomes. **A** Representative electron micrograph of a large autolysosome (AL) fused with two smaller ALs (representative of two independent experiments). **B**, **C** Representative electron micrograph of ApoExo-size vesicles in a large autolysosome (AL) (representative of two independent experiments). Arrows in (**B**) represent invagination of the inner leaflet of the AL. **D** Representative electron micrograph of HUVECs serum starved (SS) for 3 h showing the release of membrane vesicles within the extracellular compartment (representative of two independent experiments). Scale bar = 500 nm unless specified otherwise.

Because serum starvation induced a sequential activation of autophagy and apoptosis in endothelial cells, we next aimed to characterize their respective roles in the biogenesis of ApoExos and the release of immunogenic LG3. We used electron microscopy to characterize the kinetics of ultrastructural changes occurring in endothelial cells exposed to serum starvation for 1, 2, and 3 h. Under normal conditions, endothelial cells displayed well-defined uncondensed membrane-bound nuclei (Fig. 2A), and autophagosomes were uncommon in cells maintained in normal culture conditions. Serum starvation for 1 h induced a significant increase in the cytoplasmic surface area occupied by autophagosomes and autolysosomes (Figs. 2B, S3A). After 2 h of serum starvation, fusion events between autophagosomes and lysosomes became evident (Figs. 2B, S3B). Serum starvation for 3 h was associated with a further increase in the cytoplasmic surface occupied by autolysosomes as well as a reduction in the surface area occupied by autophagosomes (Figs. 2B, C, S3C). Late-stage multilamellar autophagosomes were identified within autolysosomes in association with electron-dense material (Fig. 2B). The cell surface area occupied by multivesicular bodies (MVBs) increased between 1 and 2 h of serum starvation followed by a significant decrease at 3 h (Figs. 2B, C, S3). Fusion events between MVBs and lysosomes were also identified after 2 h of serum starvation (Fig. 2B), suggesting that MVB-lysosome fusion also contributes to the formation of large autolysosomes visible in endothelial cells after 3 h of serum starvation. These large autolysosomes (2–4 µm) filled a large portion of the cytoplasm, leading to compression of the nucleus (Figs. 3, S4). Electron microscopy suggested that these large autolysosomes could also originate from a fusion of multiple autolysosomes (Fig. 3A). Murine endothelial cells from WT mice serum-starved for 9 h presented similar ultrastructural findings (Fig. S5A).

One of the most striking findings was the presence of an array of membrane vesicles of different sizes within these large autolysosomes, including sizes within the range of ApoExos (50–150 nm) (Figs. 2B, S4). Invagination of the inner side of the autolysosome membrane was also noted (Figs. 3B, C, S4D, E), raising the possibility that inward budding of autolysosome membranes could contribute to the biogenesis of membrane-bound vesicles (Figs. 3B, C, S4D, E). Additionally, contact sites and fusion events between large autolysosomes and the plasma membrane leading to the release of membrane vesicles in the extracellular space were also noted on several occasions (Figs. 3D, S4A). Electron microscopy also revealed several areas of cytoplasmic thinning between large autolysosomes and the cell membrane (Figs. 3D, S4).

### ApoExos are formed within LAMP2+ autolysosomes

**Fig. 4:**
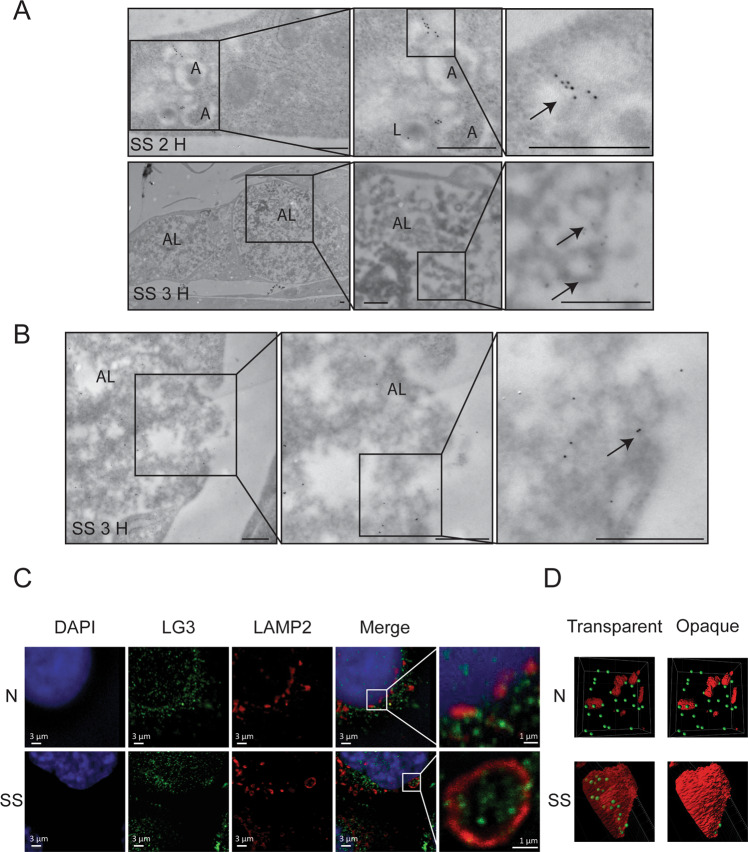
LG3 is located within large autolysosomes. **A** LG3 immunogold labeling in HUVECs serum-starved (SS) for 2 h or 3 h showing the presence of LG3 in an autophagosome (A, arrow), fused compartments (arrow), and large autolysosomes (AL, arrow). **B** Electron micrograph of HUVECs serum starved (SS) for 3 h showing LG3 immunogold labeling in a large autolysosome (AL) releasing its content in the extracellular compartment (representative of one experiment). **C** Confocal microscopy of HUVECs exposed to normal (N) or SS for 3 h (blue, DAPI; green, perlecan/LG3; and red, LAMP2) (representative of three independent experiments). **D** Representative 3D reconstruction of LAMP2+ vacuoles with transparent or opaque surfaces using Imaris software of HUVECs exposed to normal (N) or SS for 3 h (green, perlecan/LG3; and red, LAMP2). Scale bar = 500 nm unless specified otherwise.

We evaluated whether the ApoExo markers, LG3 and LAMP2, were present on membrane vesicles identified within large autolysosomes found in serum-starved endothelial cells. Using immunogold labeling, we identified LG3 in areas of fusion between autophagosomes and lysosomes (Fig. 4A) as well as on membrane vesicles present inside large autolysosomes (Fig. 4A, B). Immunogold labeling also identified LG3 on extracellular membrane vesicles released following autolysosome fusion with the cell membrane (Fig. 4B). Confocal fluorescence microscopy also confirmed the presence of LG3 within some but not all LAMP2-positive large autolysosomes in endothelial cells starved for 3 h. These results suggested that lysosomes contribute to the biogenesis of LG3-positive membrane vesicles (Figs. 4C, S6).

Under normal conditions, human endothelial cells displayed small LAMP2-positive perinuclear cytoplasmic vacuoles without LG3 colocalization (Figs. 4C, D, S6). In contrast, serum starvation led to the formation of double LG3+/LAMP2+ large vacuoles (3 µm) (Figs. 4C, D, S6). Similar findings for LG3 immunogold labeling were observed in serum-starved primary murine endothelial cells (mECs), suggesting conserved mechanisms of ApoExo secretion by human and murine endothelial cells (Fig. S5B). Collectively, these results suggested a role for autolysosomes in the biogenesis of ApoExos.

### Relative contributions of autophagy and caspase-3 activation to the biogenesis and immunogenicity of ApoExos

**Fig. 5:**
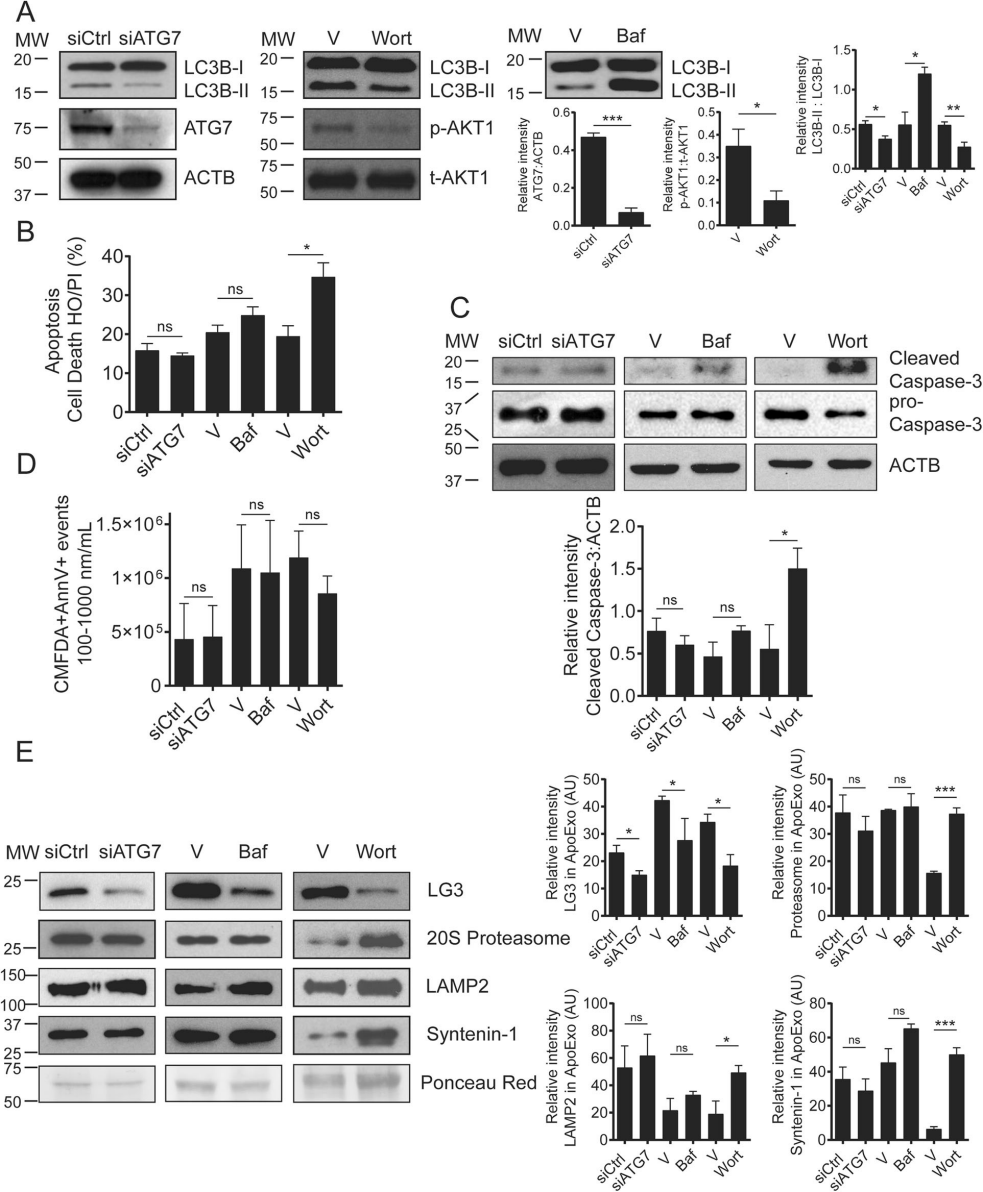
Autophagy regulates the loading of LG3 on apoptotic exosome‐like vesicles (ApoExos). **A** Representative immunoblots and densitometric analysis of LC3, ATG7, phospho-AKT1, and total AKT1 from serum-starved (SS) HUVECs transfected with siCtrl or siATG7 or exposed to vehicle (V), wortmannin (100 nM; Wort), or bafilomycin A1 (20 nM, Baf). β-actin (ACTB) was used as a loading control. *n* = 3 for each condition. **B** HO/PI staining of apoptosis in SS HUVECs transfected with siCtrl or siATG7 or exposed to V, Baf, or Wort. *n* = 3–5 for each condition. ns (nonsignificant). **C** Immunoblots and densitometric analysis of cleaved caspase-3 in SS HUVECs transfected with siCtrl or siATG7 or exposed to V, Baf, or Wort. β-actin (ACTB) was used as a loading control. *n* = 3–5 for each condition. **D** Small particle flow cytometric quantifications of CMFDA+ AnnexinV+ ApoExos detected in the supernatant of HUVECs serum starved (SS) for 4 h and transfected with siCtrl or siATG7 or exposed to V, Baf, or Wort. *n* = 3 for each condition. **E** Representative immunoblots and densitometric analysis of LG3, 20S proteasome, LAMP2, and synthenin-1 in ApoExos purified from HUVECs serum starved (SS) for 4 h and transfected with siCtrl or siATG7 or exposed to V, Baf, or Wort. Ponceau red was used as a loading control. *n* = 3–5 for each condition. *P* values were obtained by the unpaired *t* test (*P < 0.05; **P < 0.01; ***P < 0.001). All values are expressed as the mean ± SEM.

We next sought to evaluate the respective contributions of autophagy and caspase-3 activation to the biogenesis of ApoExos. We used ATG7 silencing, bafilomycin A (Baf), or wortmannin to block the early and late stages of autophagy in serum-starved endothelial cells. ATG7 silencing and wortmannin both significantly decreased LC3II/I ratios (Fig. 5A). Baf is a potent inhibitor of vacuolar H+ ATPase, which controls lysosome pH [22–24], and Baf inhibits autophagic flux [25, 26]. In the present study, Baf treatment increased LC3II/I ratios in serum-starved endothelial cells (Fig. 5A). ATG7 silencing and Baf treatment did not influence the levels of necrosis, apoptosis, or caspase-3 cleavage in serum-starved endothelial cells (Figs. 5B, C, S7A). However, wortmannin increased apoptotic features and levels of caspase-3 cleavage after 3 h of serum starvation (Fig. 5B, C). ATG7 silencing, Baf treatment, and PI3K inhibition did not modulate the secretion of extracellular vesicles (<800 nm) (Figs. 5D, S7B). We next evaluated lysosome and ApoExo markers in membrane vesicles produced by endothelial cells exposed to serum starvation and treated with siATG7, Baf, or wortmannin (Fig. 5E). ApoExos obtained from siATG-, Baf- or wortmannin-treated cells showed lower LG3 levels than controls. However, the levels of the 20S proteasome, another ApoExo marker, were not modulated by siATG7 or Baf treatment. The LAMP2 lysosomal marker was also unaltered by siATG7 and Baf treatment. Treatment with wortmannin significantly increased proteasome and LAMP2 levels, which was consistent with higher levels of apoptosis and caspase-3 cleavage in wortmannin-treated cells. Similar results were obtained for LG3 levels after treatment of mECs with Baf, suggesting conserved mechanisms of ApoExo release in mice and human endothelial cells (Fig. S7C–E). These results suggested that the autophagic process selectively regulates the expression of LG3 within ApoExos without altering their biogenesis and release.

**Fig. 6:**
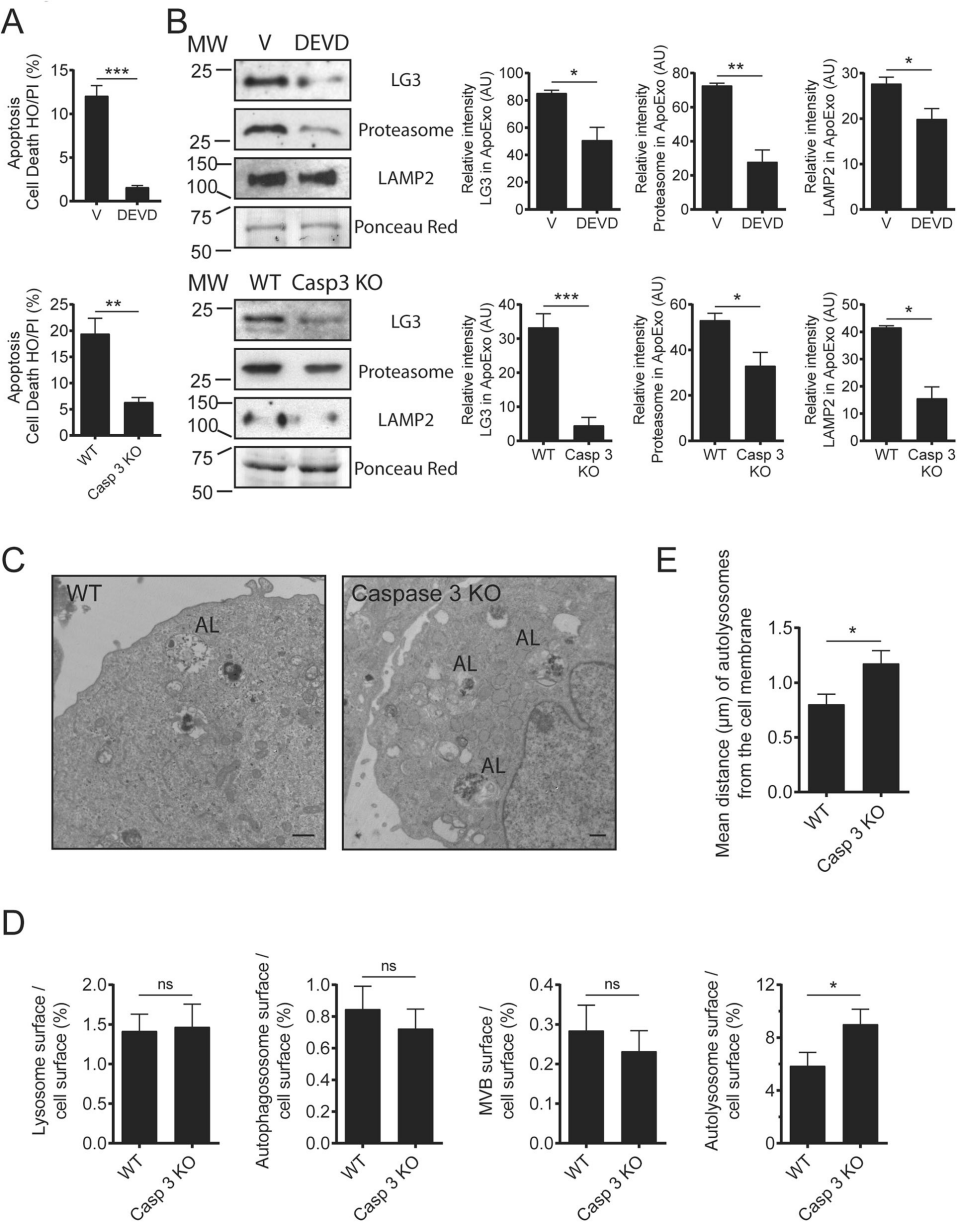
Caspase-3 regulates the release of apoptotic exosome-like vesicles (ApoExos). **A** Hoechst 33342 and propidium iodide (HO/PI) staining of apoptosis serum-starved (SS) HUVECs exposed to vehicle (V) or DEVD as well as wild-type (WT) or caspase-3 knockout (Casp3 KO) murine endothelial cells (mECs). *P* values were obtained by the unpaired *t* test. *n* = 3 for V or DEVD treatments, and *n* = 7 for WT and Casp3 KO. **B** Representative immunoblots and densitometric analysis of LG3, 20S proteasome, and LAMP2 in ApoExos purified from HUVECs serum starved (SS) for 4 h and exposed to V or DEVD as well as in ApoExos derived from Casp3−/− and wild-type mECs serum starved (SS) for 9 h. Ponceau red was used as a loading control. *P* values were obtained by the unpaired t test. *n* = 4 for each condition. **C** Representative electron micrograph of WT or Casp3−/− mECs serum-starved (SS) for 9 h showing autolysosomes (AL, representative of two independent experiments). Scale bar = 500 nm. **D** Quantification of the cell surface occupied by lysosomes, autophagosomes, multivesicular bodies, and autolysosomes in WT or Casp3−/− mECs SS for 9 h. *P* values were obtained by the unpaired *t* test. *n* = 50 cell profiles from two independent experiments for each condition. ns (nonsignificant). **E** Quantification of the distance between the autolysosomes and the plasma membrane in WT or Casp3−/− mECs SS for 9 h. P values were obtained by the Mann–Whitney test. *n* = 50 cell profiles from two independent experiments for each condition. *P < 0.05; **P < 0.01; ***P < 0.001. All values are expressed as the mean ± SEM.

We then used caspase-3−/− mice or caspase inhibitors (DEVD or ZVAD) to assess the importance of caspase activation in the biogenesis and release of ApoExos. ApoExos obtained from serum-starved caspase-3−/− mECs and DEVD-treated cells showed lower proteasome, LAMP2, and LG3 levels (Figs. 6A, B, S8A). We then used electron microscopy to evaluate autophagosome, lysosome, and MVB dynamics in caspase-3−/− mECs exposed to serum starvation (Fig. 6C). The cell surface occupied by autolysosomes was significantly higher in caspase-3−/− cells than in WT cells (Fig. 6D), while the surface occupied by autophagosomes and MVB cells was not significantly different. The distance between autolysosomes and the cell membrane was significantly greater in caspase-3−/− mECs than in wild-type controls (Fig. 6E). Similar results were obtained in serum-starved HUVECs exposed to ZVAD (Fig. S8B). Collectively, these results suggested that caspase-3 does not regulate early fusion between lysosomes, MVBs, and autophagosomes but instead plays a major role in regulating fusion events between autolysosomes and the cell membrane, thus regulating the release of ApoExos.

**Fig. 7:**
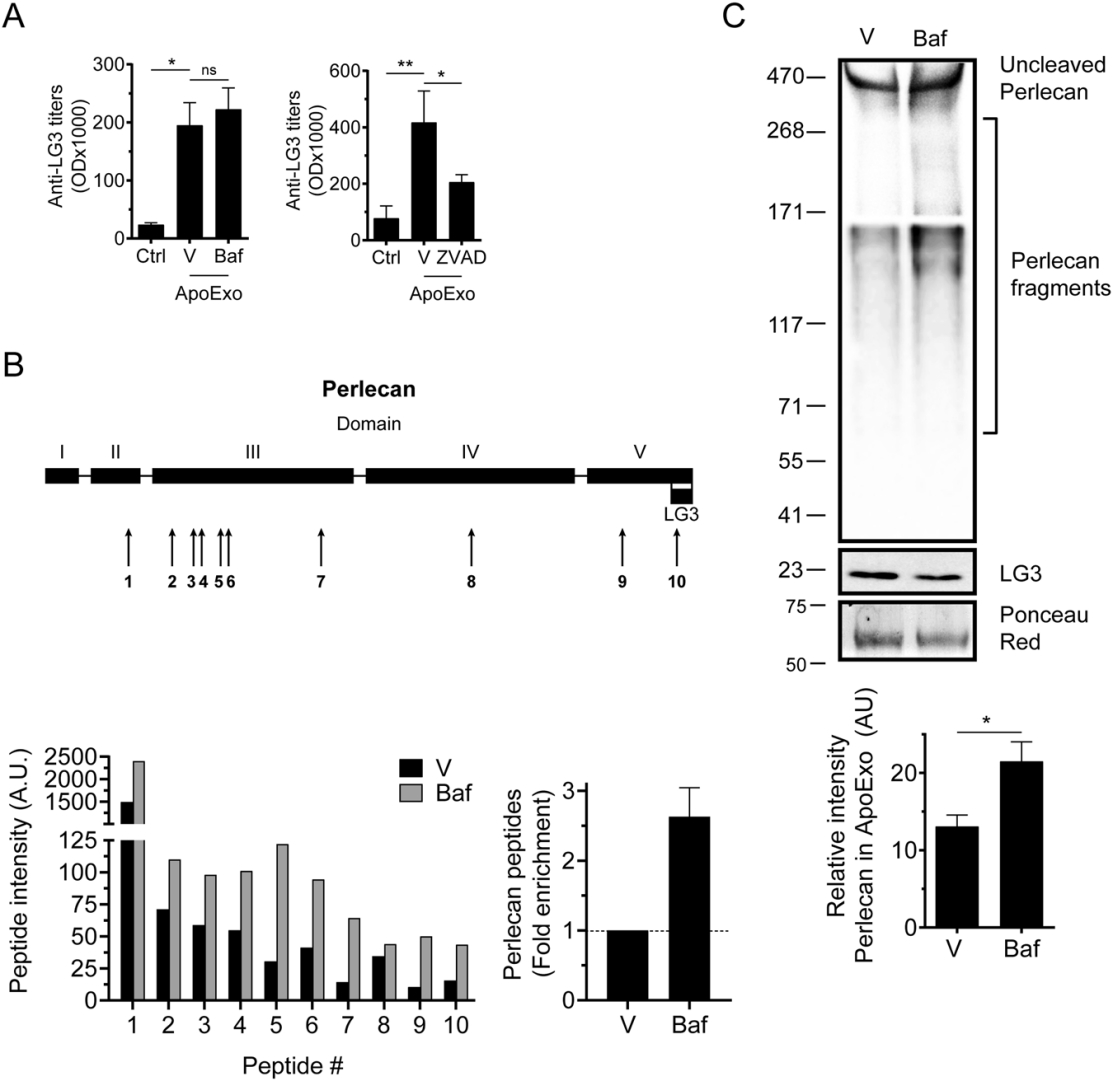
Autophagy controls the cleavage of perlecan to LG3 fragments in apoptotic exosome-like vesicles (ApoExos). **A** Serum anti-LG3 IgG titers in allografted mice after 3 weeks of intravenous injections with vehicle (Ctrl) or ApoExos produced by serum-starved mECs treated with vehicle (ApoExo V), bafilomycin A1 (20 nM, ApoExo Baf), or ZVAD-FMK (50 μM, ApoExo ZVAD). *P* values were obtained by one-tailed unpaired *t* test. ns (nonsignificant). *n* = 7 mice for V and Baf injections, and *n* = 9 mice for V and ZVAD-FMK injections. **B** Representation of the abundance of perlecan peptides in ApoExos from serum-starved mECs treated with vehicle (V) or bafilomycin A1 (20 nM, Baf). Fold enrichment of perlecan peptide intensity in ApoExos from bafilomycin-treated cells compared to ApoExos from vehicle-treated cells. **C** Representative immunoblots and densitometric analysis of perlecan and LG3 fragments in ApoExos purified from HUVECs serum starved (SS) for 4 h in the presence of V or Baf. Ponceau red was used as a loading control. *P* values were obtained by the unpaired *t* test. *n* = 3 for each condition. *P < 0.05; **P < 0.01; ***P < 0.001. All values are expressed as the mean ± SEM.

ApoExos are known to exert an immunogenic function, at least in part, through the release of LG3, leading to the production of anti-LG3 antibodies that, in turn, aggravate rejection episodes in mice and humans [12]. To determine the relative contributions of autophagy and caspases to the immunogenicity of ApoExos, we purified ApoExos produced by murine endothelial cells exposed to Baf, ZVAD, or the control (Fig. S7D, E). ApoExo preparations were then injected into mice transplanted with an allogeneic aortic allograft, and the production of anti-LG3 antibodies was monitored. Intriguingly, ApoExos from bafilomycin-treated cells, which were devoid of the LG3 fragment, triggered the production of anti-LG3 antibodies to levels similar to those of controls. In contrast, the production of anti-LG3 antibodies was significantly reduced in mice injected with ApoExos from ZVAD-treated cells (Fig. 7A). These results confirmed the central role of caspases in regulating the release of immunogenic ApoExos. However, the immunogenic activity of ApoExos produced by bafilomycin-treated cells suggested the possibility that ApoExos could bear a variety of perlecan fragments containing the LG3 motif, therefore contributing to anti-LG3 production. To evaluate this possibility, murine endothelial cells were serum-starved in the presence of bafilomycin or vehicle control. ApoExos were purified and subjected to proteomic analysis for the identification of perlecan-derived peptides. Proteomic analysis identified ten perlecan peptide sequences in control ApoExos or ApoExos from bafilomycin-treated cells. Nine of these peptides were identified from regions of the perlecan N-terminus to the LG3 fragment. Using PEAKS X to analyze relative protein abundance, all identified peptides were enriched in ApoExos derived from bafilomycin-treated cells (Fig. 7B). These results suggested that the bafilomycin-treated ApoExos were likely bearing perlecan fragments of higher molecular weight, which may be a reflection of reduced proteolysis in the presence of bafilomycin. Immunoblot analysis confirmed this hypothesis. ApoExos from bafilomycin-treated cells showed decreased levels of the LG3 fragment but increased levels of higher molecular weight fragments bearing the LG3 motif (Fig. 7C). Collectively, these results demonstrated the central role of caspase-3 in regulating the release of immunogenic ApoExos formed within large autolysosomes, while autophagy controls the degradation of perlecan into low molecular weight fragments, including LG3. However, degradation of perlecan in autolysosomes is dispensable for the immunogenic activity of ApoExos and the production of anti-LG3 antibodies.

## Discussion

Mounting evidence associates graft rejection and ischemia–reperfusion injury with the presence of autoantibodies reactive to components of endothelial cells or apoptotic cells in general [12, 24–28]. These autoantibodies, whose normal role is likely to favor the clearance of apoptotic cells, can increase allograft vascular injury and negatively impact allograft survival [11, 29–31]. ApoExos derived from endothelial cells foster the formation of these autoimmune responses, including the formation of anti-LG3 antibodies [10–13]. Classical apoptotic bodies also released downstream of caspase activation do not foster autoantibody production and inflammation [10, 11]; in contrast, they have been shown to exert anti-inflammatory activity in various contexts. Classical apoptotic bodies are released through blebbing of the apoptotic cell membrane. However, the mechanisms of ApoExo biogenesis remain to be delineated. In the present study, we identified autolysosomes and caspase-3 as important regulators of ApoExo maturation and release. We demonstrated that endothelial cells under nutrient depletion promoted an autophagic response that favored lysosome and MVB interactions, leading to the formation of membrane vesicles within large autolysosomes. We also identified caspase-3 as a key regulator of fusion events between autolysosomes and the plasma membrane, therefore controlling the release of ApoExos. These results supported previous studies showing enrichment of LG3 and the proteasome in exosome-like vesicles derived from apoptotic cells [16, 32]. These findings also further supported the notion that apoptotic cells produce different types of extracellular vesicles, some of which are aimed at alerting the immune system and triggering a proinflammatory response [11, 17].

In the present study, the use of electron microscopy and immunogold labeling was instrumental in characterizing the respective roles of autophagosomes, MVBs, and lysosomes in the formation of ApoExos. Large LAMP2+ autolysosomes, containing components of autophagosomes and MVBs, were found to contribute to the formation of membrane vesicles bearing the LG3 autoantigen. These results were in line with a growing body of evidence suggesting a role for autophagy in unconventional protein secretion, a process referred to as secretory autophagy [33]. Secretory autophagy controls the secretion of proteins that lack peptide signals, but it can also behave as an alternative pathway to promote the trafficking of proteins toward the plasma membrane. Secretory autophagy has been shown to develop in association with inflammation and tissue remodeling [34, 35]. In some instances, extracellular matrix components can be secreted in an autophagy-dependent manner. Indeed, knockdown of ATG7 in pancreatic stellate cells decreases the secretion of extracellular matrix components, especially fibronectin [36].

Recent evidence suggests that lysosomes and organelles of the autophagy system, both traditionally viewed as degradative organelles, also play important roles in unconventional secretion pathways. Secretory autophagy, resulting from the fusion of autophagosomes or amphisomes, to the cell membrane is central to the release of various mediators including ILB1, HMGB1, and TGFB [37–39]. Secretory lysosomes also contribute to non-classical secretion mechanisms by fusing the cell membrane, therefore releasing their content extracellularly [40]. Lysosome exocytosis may also contribute to the release of exosomes [41, 42]. Autolysosomes form from complex fusion events between autophagosomes, multivesicular bodies and lysosomes in starved cells. In this context, the prevailing view is that the intraluminal content of autolysosomes is destined to degradation rather than secretion. Our results challenge this view and demonstrate that autolysosomes, like autophagosomes and lysosomes, can fuse with the cell membrane to release their content extracellularly. Our results also identify caspase-3 as an important regulator of fusion events between autolysosomes and the cell membrane, therefore controlling the release of ApoExos.

Cellular stress induced by starvation contributes to autophagosome formation and to transcriptionally-mediated increases in lysosome numbers [43]. Recent work has shown that starvation regulates lysosome membrane turnover as well as lysosome size and function [44]. Glucose starvation induces lipidation of the autophagy-associated LC3 protein onto lysosomal membrane and microautophagy-dependent formation of intraluminal vesicles. Invagination of lysosome membranes during microautophagy contributes to the translocation of Hsc70-targeted entities within the lysosome [45]. The LC3-conjugation machinery and neutral sphingomyelinase 2 have recently been shown to control extracellular vesicle cargo loading and secretion in starved cells [7]. Whether microautophagy and LC3 lipidation also contribute to membrane vesicle formation within autolysosomes will be the scope of future studies.

Interfering with autophagosome formation using ATG7 silencing or inhibition of lysosomal acidification with bafilomycin decreased LG3 fragment secretion in ApoExos without modulating the secretion of other ApoExo markers, such as the 20S proteasome or LAMP2. These findings suggested that autophagy-independent pathways control the loading of the 20S proteasome onto ApoExos, and they also highlighted the importance of the autophagic process in perlecan degradation and the loading of the LG3 fragment within exosome-like vesicles. The induction of anti-LG3 autoantibodies by ApoExos from bafilomycin-treated cells was another clue to the importance of the autophagic process in perlecan degradation. Proteomic analysis of ApoExos originating from bafilomycin-treated cells highlighted the presence of perlecan motifs at the N-terminus of LG3. Immunoblot analysis confirmed the presence of higher molecular weight forms of perlecan bearing the LG3 motif in ApoExos originating from bafilomycin-treated cells. Unlike perlecan, the LG3 fragment does not possess a signal peptide, and it stems from cleavage of native perlecan by lysosomal proteases, such as cathepsin L [23]. The present results identified the autophagic process as an important contributor to the generation of the LG3 fragment and its loading onto ApoExos.

Although the present results highlighted an important role for autolysosomes in the biogenesis of ApoExos, they also stressed the importance of caspase-3 in the fusion of autolysosomes with the cell membrane, leading to ApoExo secretion. In our system, caspase inhibition and caspase-3 deficiency prevented the release of ApoExos, and they were associated with the cytoplasmic accumulation of autolysosomes. The size of autolysosomes and the mean distance between autolysosomes and the cell membrane increased in caspase-3-deficient cells and pancaspase-inhibited cells. Mounting evidence suggests that caspase activity modulates membrane dynamics involved in the regulation of extracellular vesicle release. Activation of noncanonical caspase-4/-5 inflammasomes has been implicated in EV secretion in human macrophages [46]. We also previously described the secretion of autophagosome constituents by endothelial cells downstream of caspase-3 activation [15]. More recently, caspase-3 activation in hepatocytes has been found to regulate the release of EVs containing CD40L [47].

**Fig. 8:**
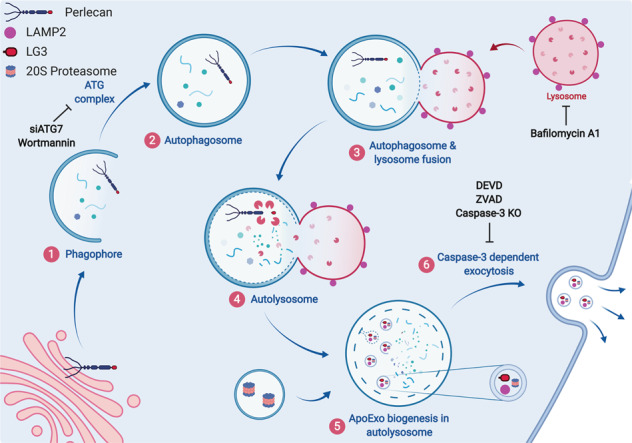
Autolysosomes control the biogenesis of apoptotic exosome-like vesicles (ApoExos), and caspase-3 controls their release. Autophagy plays a central role in the degradation of perlecan and the loading of perlecan fragments into ApoExos forming within large autolysosomes. Caspase-3 activation regulates fusion between autolysosomes and the plasma membrane, thereby controlling the secretion of immunogenic ApoExos. The schematic was created with BioRender.com.

Collectively, these results uncovered an important role for autolysosomes in the biogenesis of membrane vesicles bearing LG3 and for autophagy in the intracellular degradation of perlecan and loading of perlecan fragments onto ApoExos (Fig. 8). These findings also highlighted a central role for caspase-3 in membrane fusion between autolysosomes and the cell membrane, therefore controlling the release of ApoExos. A better understanding of the molecular pathways controlling the production of ApoExos at sites of vascular injury opens new possibilities for controlling the propagation of inflammation.

## Supplementary information

Source data 1

Supplementary data

Figure S1

Figure S2

Figure S3

Figure S4

Figure S5

Figure S6

Figure S7

Figure S8

aj-checklist

## Data Availability

Data for Fig. 7 are provided in Source Data 1.
