# Supplementary material for: Autolysosomes and caspase-3 control the biogenesis and release of immunogenic apoptotic exosomes
Source: Cell Death Dis. 2022 Feb 11;13(2):145. doi: 10.1038/s41419-022-04591-5 (PMC8837616; doi:10.1038/s41419-022-04591-5)
Supplement: Supplementary file 2 — Supplementary data [file 41419_2022_4591_MOESM2_ESM.pdf]

Supplementary Materials for

Autolysosomes and caspase-3 control the biogenesis and release of immunogenic apoptotic exosomes

Déborah Beillevaire[1,2,5], Francis Migneault[1,2], Julie Turgeon[1,2], Diane Gingras[4], Annie Karakeussian Rimbaud[1,2], Geneviève Marcoux[7], Crysta Spino[1], Nicolas Thibodeau[1], Eric Bonneil[6], Pierre Thibault[2,6], Éric Boilard[2,7], Mélanie Dieudé[1,2,3], and Marie-Josée Hébert[1,2,5].

[1]Centre de recherche, Centre hospitalier de l'Université de Montréal (CRCHUM) and Université de Montréal, Montréal, QC, Canada

[2]Canadian Donation and Transplant Research Program (CDTRP), Canada.

[3]Département de microbiologie, infectiologie et immunologie, Faculté de Médecine, Université de Montréal, Montréal, QC, Canada.

[4]Département de pathologie et biologie cellulaire, Université de Montréal, Montréal, QC, Canada.

[5]Département de médecine, Université de Montréal, Montréal, QC, Canada.

[6]Institute for Research in Immunology and Cancer (IRIC), Montréal, QC, Canada.

[7]Centre de Recherche, Centre Hospitalier Universitaire (CHU) de Québec-Université Laval, Département de microbiologie et immunologie Québec, QC, Canada.

Running title: **Autolysosomes and caspase-3 regulate maturation and release of ApoExos**

Corresponding author

*Dr. Marie-Josée Hébert, marie-josee.hebert@umontreal.ca

CRCHUM, 900 St. Denis Street, Pavilion R, Room R12.412, Montreal, QC, Canada H2X 0A9.
Telephone: (514) 890-8000 Extension 28479, Fax: (514) 412-7944.

Figure S1. Characterization of the apoptotic exosome-like exosome (ApoExos). Immunoblots and densitometric analysis of GM130, LAMP2, Tubulin, TSG101, CD82, Syntenin-1, 20S proteasome, LG3, TCTP, and histone H3 in apoptotic bodies or apoptotic exosome-like vesicles purified from HUVECs SS for 4h. P values obtained by unpaired t-test, n ≥ 3 for each condition.

Figure S2. Apoptosis but not necrosis induces the secretion of LG3 within apoptotic exosome-like vesicles (ApoExos). (**A**) Evaluation by Hoescht 33342 and Propidium iodide (HO/PI) staining of apoptosis and necrosis in normal medium (N) or serum-starved (SS) HUVECs incubated alone or with H_2O_2 (Nec; necrotic condition) for 4 h. P values obtained by unpaired t-test and Mann-Whitney test. n = 10 for each condition. Scale bar: 25 μm (**B**) Representative immunoblot and densitometric analysis of cleaved caspase-3 in SS or Nec HUVECs. β-actin (ACTB) used as a loading control. P values obtained by unpaired t-test. n = 3 for each condition. (**C**) Evaluation by Annexin V (AnnV) and PI staining of apoptosis and necrosis in serum-starved (SS) HUVECs incubated alone or with H_2O_2 (Nec) for 4 h. P values obtained by Mann-Whitney test. n = 6 for each condition. (**D**) Representative immunoblot and densitometric analysis of LG3 and LAMP2 in ApoExos purified from apoptotic (Apo) or necrotic (Nec) HUVECs. Ponceau red is used as a loading control. P values obtained by unpaired t-test. n = 3-5 for each condition. All values are expressed as mean ± SEM.

Figure S3. Serum-starved (SS) human endothelial cells display large vacuolar networks containing an array of membrane vesicles. (**A**) Representative electron micrograph of HUVECs serum-starved (SS) for 1 h showing an autophagosome (A) (representative of 2 independent experiments). (**B**) Representative electron micrographs of HUVECs SS for 2 h showing small autolysosomes (AL) in formation (representative of 2 independent experiments). (**C**)

Representative electron micrograph of HUVECs SS for 3 h showing large autolysosomes in formation closed from each other (representative of 2 independent experiments).

Figure S4 Membrane vesicles within large autolysosomes in serum-starved (SS) human endothelial cells. (**A-C**) Representative electron micrographs of HUVECs serum-starved (SS) for 3 h showing a large autolysosome (AL) in fusion with the plasma membrane and releasing its content in the extracellular compartment, a large AL near the plasma membrane, and cytoplasmic thinning between large AL and the cell membrane (representative of 2 independent experiments). (**D, E**) Representative electron micrographs of serum-starved (SS) HUVECs for 3 h showing invagination of the inner leaflet of large AL membrane (representative of 2 independent experiments).

Figure S5. LG3 is located within large autolysosomes in murine endothelial cells (mEC). (**A**) Representative electron micrographs of WT mEC serum-starved (SS) for 9 h showing autophagosomes (A), lysosomes (L), multivesicular bodies (MVB), small autolysosomes (AL) in formation, and larger AL containing membrane vesicles of different sizes (representative of 1 independent experiment). (**B**) Representative electron micrographs of WT mEC SS for 9 h showing LG3 immunogold labeling in a large AL (arrow, representative of 1 independent experiment).

Figure S6. LG3 is located within large LAMP2+ vesicular networks. Representative confocal microscopy images of normal (N) HUVECs or serum-starved (SS) HUVECs for 3 h (Blue: DAPI, Green: perlecan/LG3, Red: LAMP2) (representative of 3 independent experiments). Negative control (Ctrl -) with secondary fluorescent antibodies. Scale bar: 5 µM

Figure S7. Autophagy regulates LG3 loading onto apoptotic exosome-like vesicles (ApoExos). (A) Representative immunoblots and densitometric analysis of LC3, ATG7, phospho-AKT1, and total AKT1 from normal HUVECs (N) or serum-starved (SS) HUVECs transfected with siCtrl or siATG7 or exposed to vehicle (V), wortmannin (100 nM; Wort) or bafilomycin A1 (20 nM, Baf). β-actin (ACTB) used as a loading control. n = 3 for each condition. (**B**) Evaluation by HO/PI staining of necrosis in SS HUVECs transfected with siCtrl or siATG7 or exposed to vehicle (V), wortmannin (100 nM; Wort) or bafilomycin A1 (20 nM, Baf). P values obtained by unpaired t-test. n = 3-5 for each condition. ns (non-significant). (**C**) Representative small particles flow cytometric gates of CMFDA+AnnV+ ApoExos detected in total supernatant of 4h SS HUVECs transfected with siCtrl or siATG7 or exposed to V, Wort, or Baf. n = 3 for each condition. (**D**) Representative immunoblot of LC3 in SS murine endothelial cells (mEC) exposed to V or Baf for 9 h (representative of 2 independent experiments). (**E**) Evaluation by HO/PI staining of apoptosis (Apo) and necrosis (Nec) in SS mEC exposed to V, Baf, or ZVAD for 9 h. P values obtained by unpaired t-test. n = 2 for each condition. ns (non-significant). (**F**) Representative immunoblot of LG3 in ApoExos purified from SS mEC exposed to V and Baf or V and ZVAD for 9 h (representative of 2 independent experiments). Ponceau red is used as a loading control. All values are expressed as mean ± SEM.

Figure S8. Caspase-3 plays an important role in the fusion between the autolysosome and the plasma membrane in serum starved endothelial cells. (**A**) Evaluation by Hoescht 33342 and Propidium iodide (HO/PI) staining of necrosis in serum-starved (SS) HUVECs exposed to vehicle (V) or DEVD or in wild type (WT) or caspase-3 knock-out (Casp3 KO) murine endothelial cells (mEC). P values obtained by unpaired t-test. n=3 for V or DEVD treatments and n = 7 for WT and Casp3 KO. ns (non-significant). (**B**) Quantification of the cell surface area occupied by

autophagosome, lysosome, multivesicular bodies (MVB) and autolysosome in SS HUVECs exposed to V or ZVAD for 4 h. P values obtained by Mann-Whitney test. n = 50 cell profiles from 1 independent experiment for each condition. ns (non-significant). (**C**) Quantification of the distance between the autolysosomes and the plasma membrane in SS HUVECs exposed to V or ZVAD for 4 h. P values obtained by Mann-Whitney test. n = 50 cell profiles from 1 independent experiment for each condition. All values are expressed as mean ± SEM.
