# Supplementary figures and images for: Autolysosomes and caspase-3 control the biogenesis and release of immunogenic apoptotic exosomes
Source: Cell Death Dis. 2022 Feb 11;13(2):145. doi: 10.1038/s41419-022-04591-5 (PMC8837616; doi:10.1038/s41419-022-04591-5)

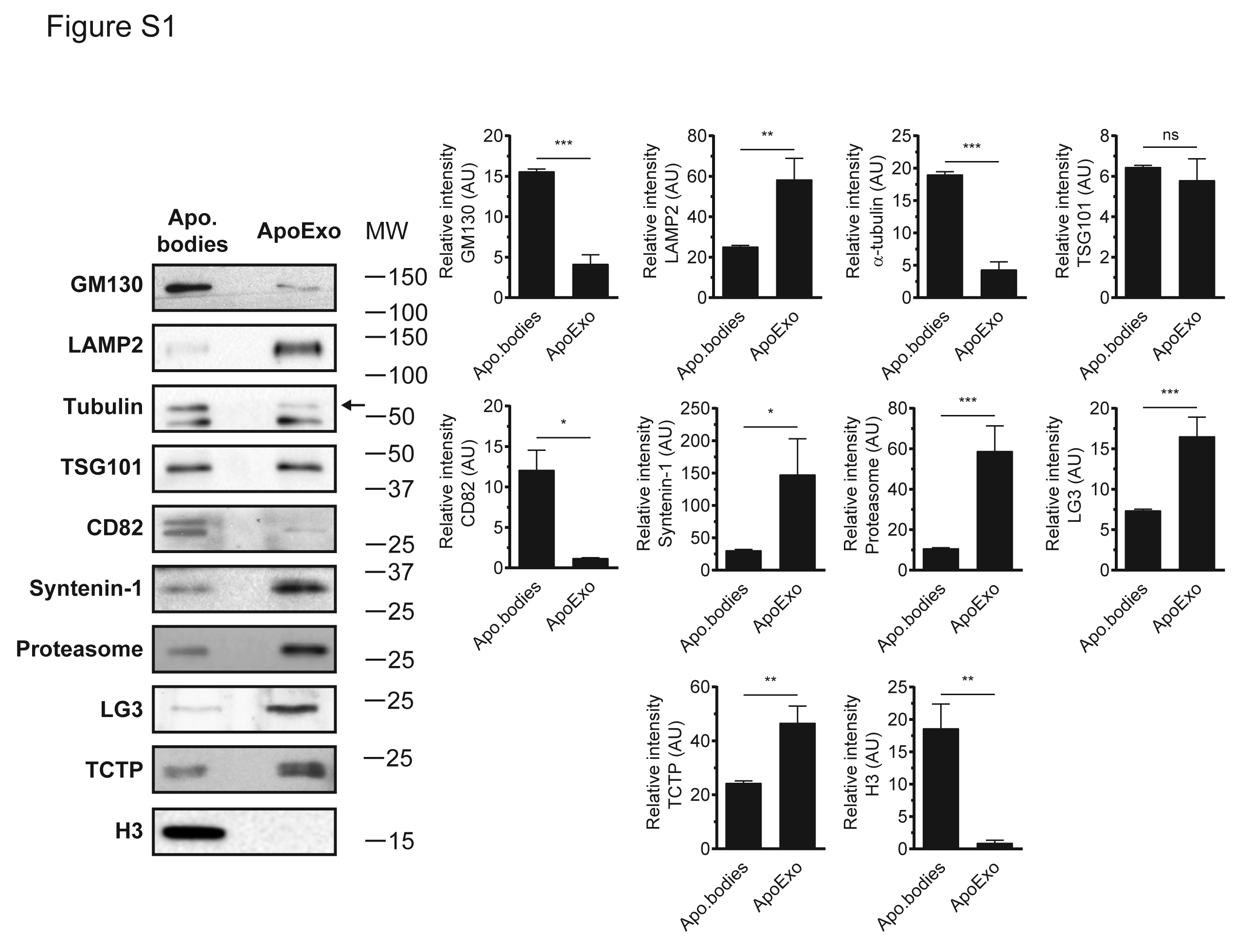

Supplement: Supplementary file 3 — Figure S1 [file 41419_2022_4591_MOESM3_ESM.tif]

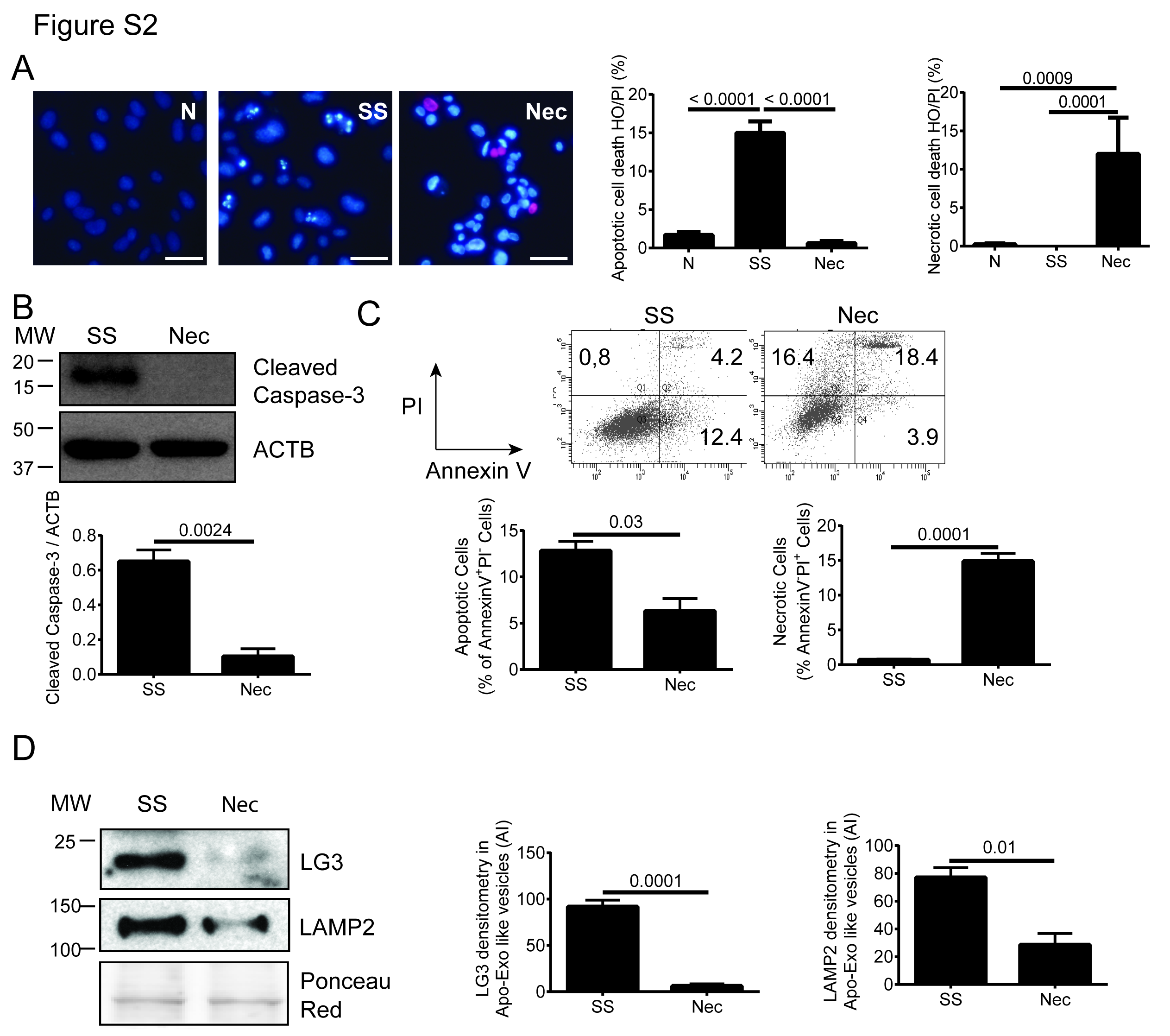

Supplement: Supplementary file 4 — Figure S2 [file 41419_2022_4591_MOESM4_ESM.tif]

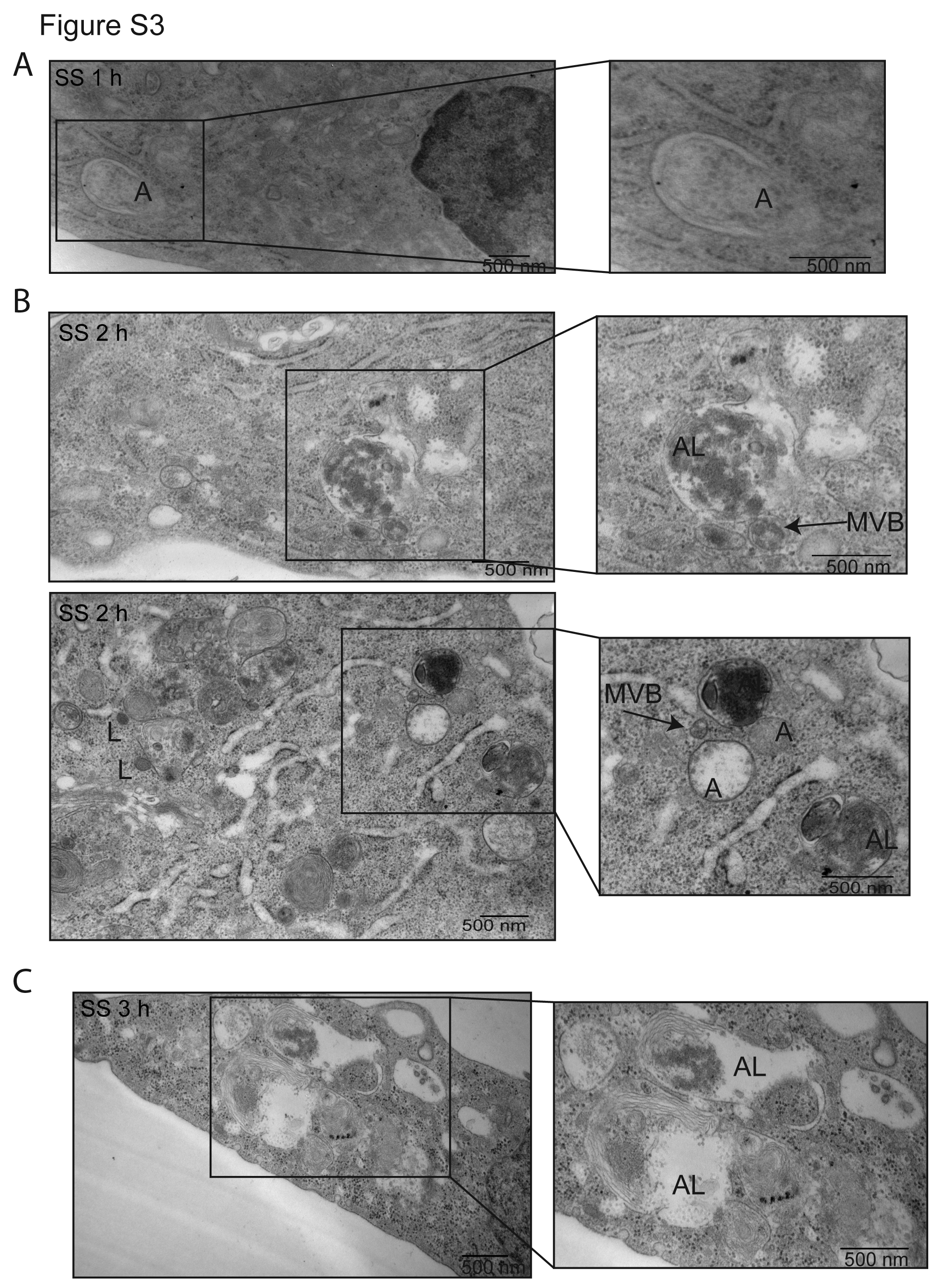

Supplement: Supplementary file 5 — Figure S3 [file 41419_2022_4591_MOESM5_ESM.tif]

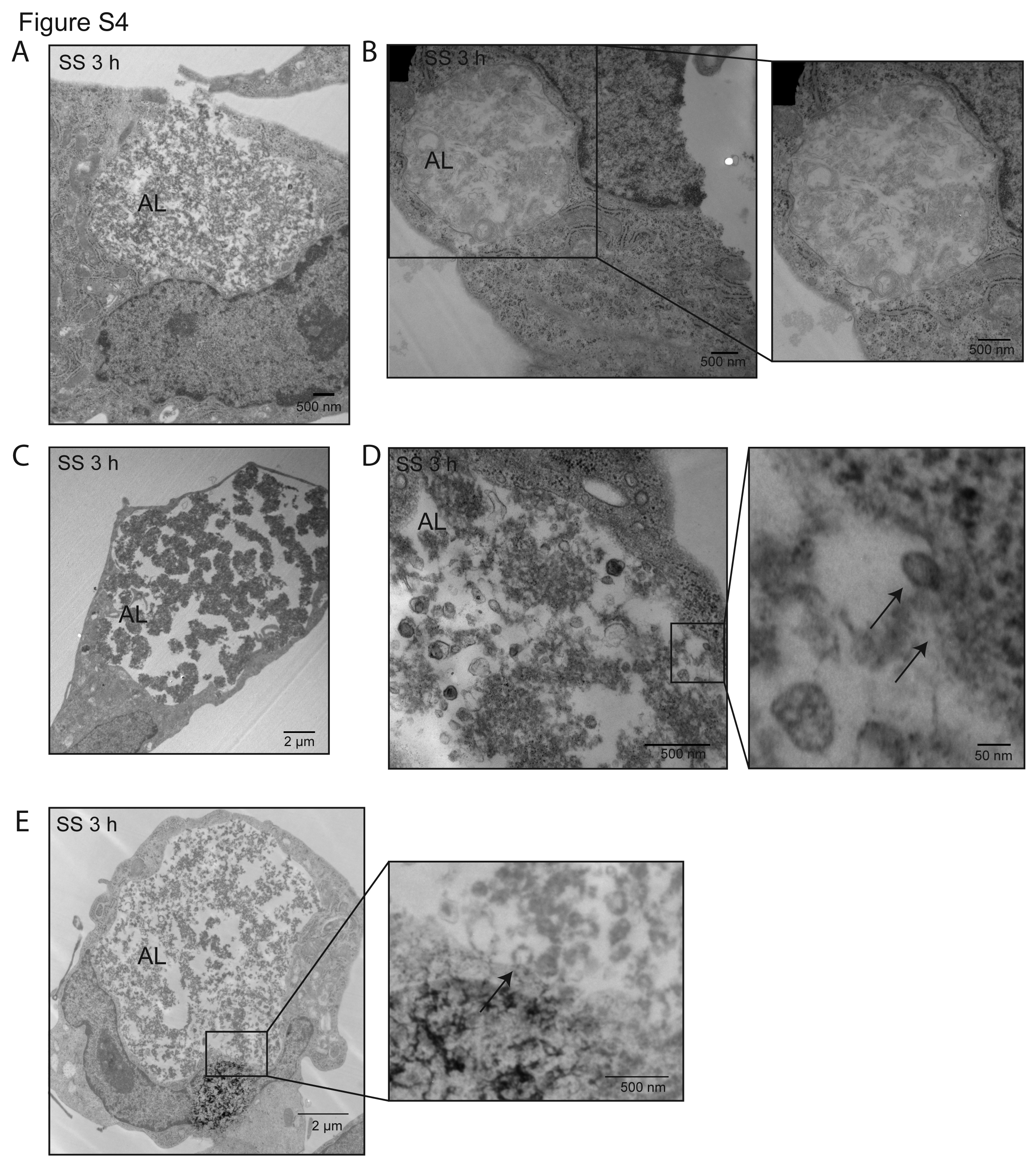

Supplement: Supplementary file 6 — Figure S4 [file 41419_2022_4591_MOESM6_ESM.tif]

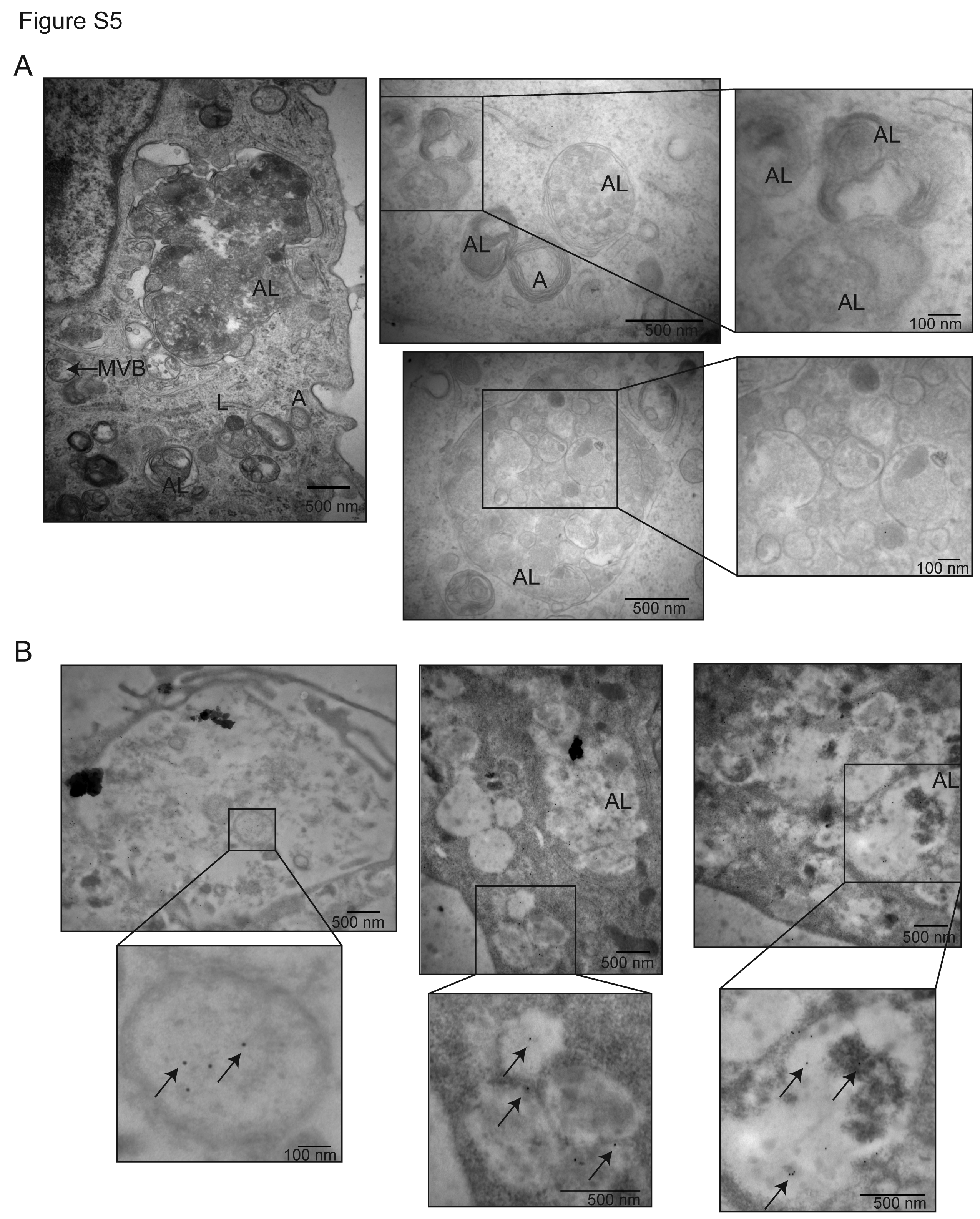

Supplement: Supplementary file 7 — Figure S5 [file 41419_2022_4591_MOESM7_ESM.tif]

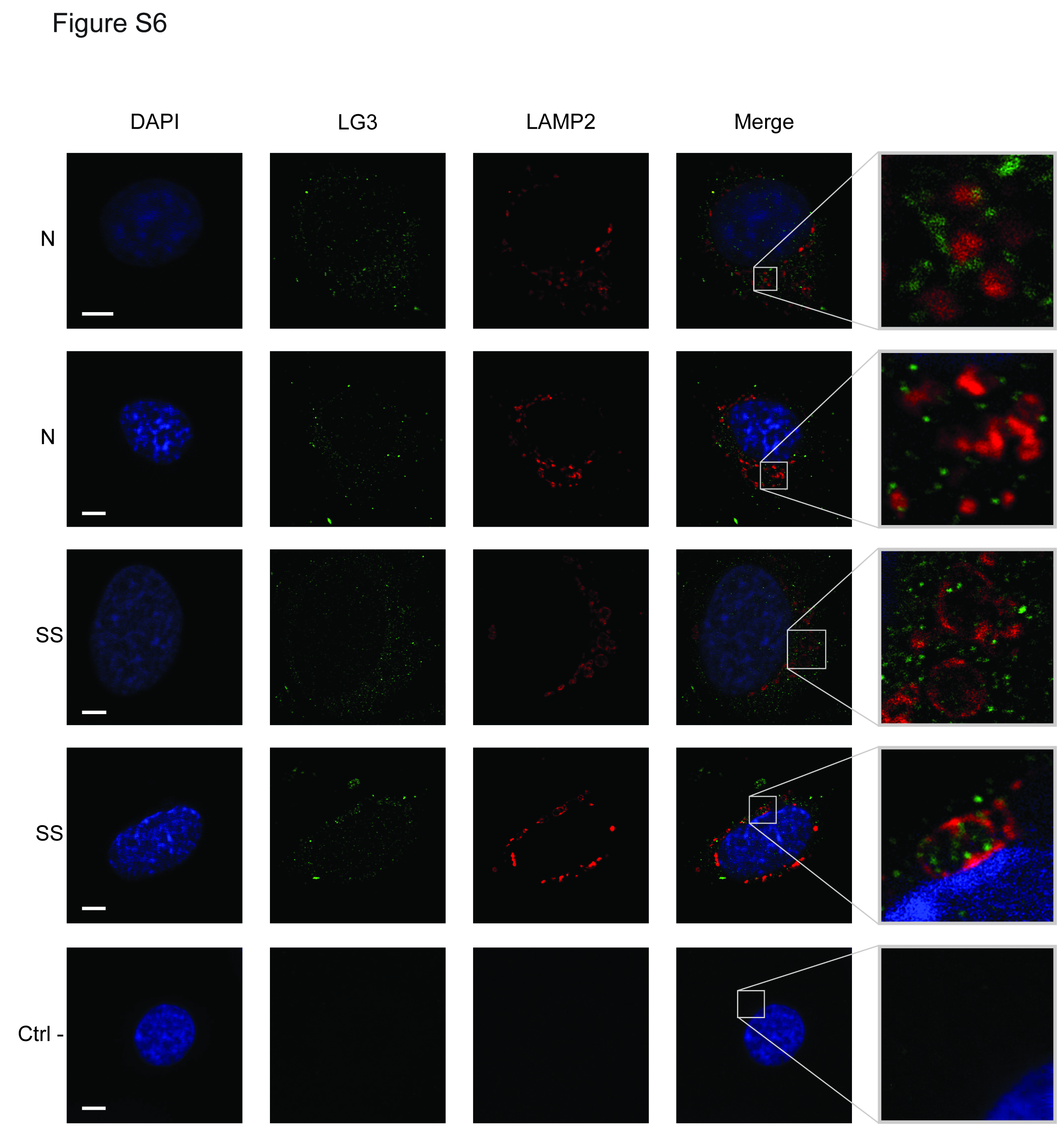

Supplement: Supplementary file 8 — Figure S6 [file 41419_2022_4591_MOESM8_ESM.tif]

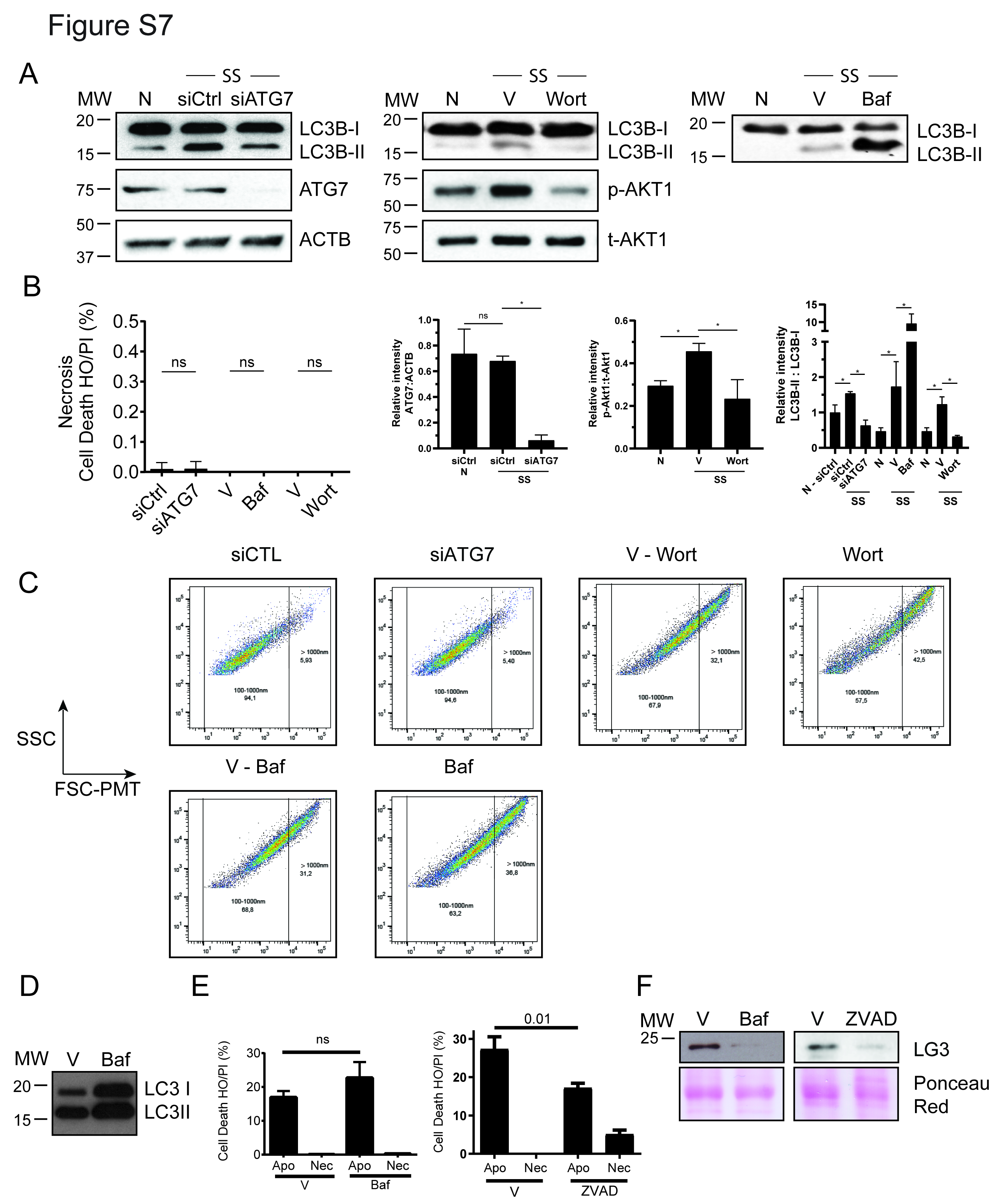

Supplement: Supplementary file 9 — Figure S7 [file 41419_2022_4591_MOESM9_ESM.tif]

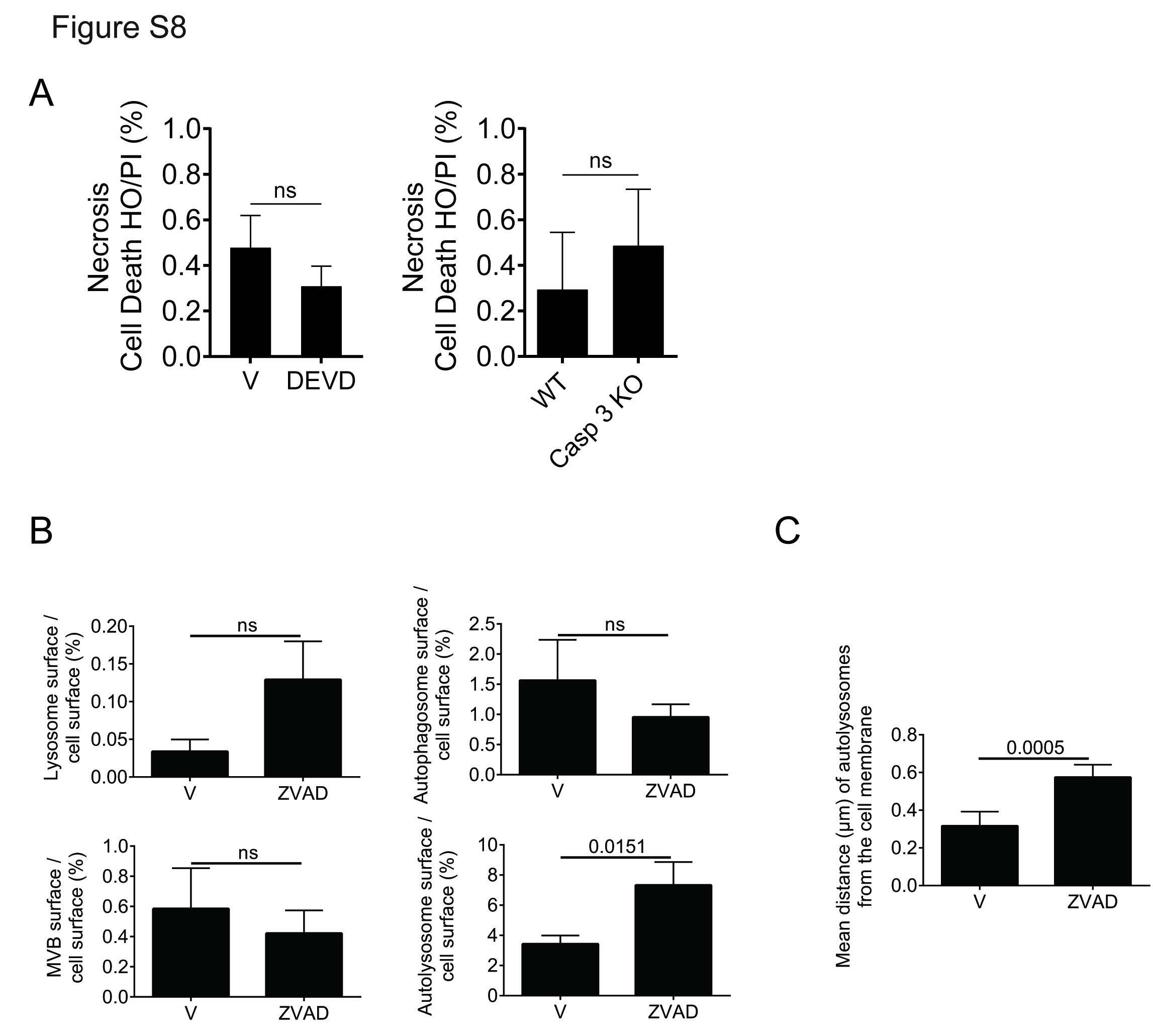

Supplement: Supplementary file 10 — Figure S8 [file 41419_2022_4591_MOESM10_ESM.tif]
